# Botanical Origin Influence on Some Honey Physicochemical Characteristics and Antioxidant Properties

**DOI:** 10.3390/foods12112134

**Published:** 2023-05-25

**Authors:** Loredana Elena Vîjan, Ivona Cristina Mazilu, Carmen Enache, Sebastian Enache, Carmen Mihaela Topală

**Affiliations:** 1 Faculty of Sciences, Physical Education and Computer Science, University of Pitesti, 1 Targu din Vale Street, 110142 Pitesti, Romania; 2 Research Institute for Fruit Growing Pitesti-Maracineni, 402 Marului Street, 117450 Maracineni, Romania

**Keywords:** Romanian honey, total sugar content, 5-hydroxymethylfurfural, polyphenols, tannins, flavonoids, DPPH radical inhibition, ATR-IR spectroscopy, chemometric analysis

## Abstract

Five types of honey (multifloral, sunflower, linden, rapeseed, and acacia), from Southern Romania, were classified using chemometrics methods coupled with IR spectroscopy. The botanical origin’s effect on the physicochemical characteristics of honey was studied to highlight the most valuable plant source of honey. Except for antioxidant activity, the moisture, ash, electrical conductivity (EC), pH, free acidity (FA), total sugar content (TSC), hydroxymethylfurfural (HMF), total phenolic (TPC), tannin (TTC), and flavonoid content (TFC) were significantly influenced by the botanical origin of the honey. The results showed that sunflower honey had the highest moisture (15.53%), free acidity (16.67 mEq kg^−1^), electrical conductivity (483.92 µS cm^−1^), phenolics (167.59 mg GAE 100 g^−1^), and flavonoids (19.00 mg CE 100 g^−1^), whereas multifloral honey presented the highest total sugar content (69.64 g Glu 100 g^−1^). The highest HMF content was found in linden honey (33.94 mg kg^−1^). The HMF contents of all tested honey were within the standard recommended limit, and they confirmed that the tested honey was free of any heat treatment. All five types of tested honey presented a safe moisture content for storage and consumption (12.21–18.74%). The honey′s free acidity was in the range of 4.00 to 25.00 mEq kg^−1^; this indicated the freshness of the samples and the absence of any fermentation processes in the tested honey. Honey with a total sugar content over 60% (except for linden honey, with 58.05 g glucose 100 g^−1^) showed the characteristic of nectar-derived honey. The elevated antioxidant activity of honey was correlated with its high moisture, flavonoids, and HMF, whereas the tannins and HMF were positively correlated with ash and electrical conductivity. The higher content of phenolics, flavonoids, and tannins was correlated with higher free acidity. The chemometric method, coupled with ATR-FTIR spectra, revealed a clear separation between linden honey from acacia, multifloral, and sunflower honey.

## 1. Introduction

Honey is a natural food produced by honeybees, *Apis melifera*, from the nectar of blossoms or exudates of trees and plants giving nectar honey or honeydews [1].

Honey production is an enzymatic process, completed with dehydration. Honeybees consume nectar and pollen as carbon and nitrogen sources, respectively, and both of these foods are subjected to gut processing, an enzymatic process of breaking down the nectar’s sugar into simple sugars (mainly glucose and fructose). The second step is to pass this nectar/sugar mixture to the younger honeybees, who convert it to honey via another enzymatic step involving three enzymes secreted by the hypopharyngeal glands of workers: alpha-glucosidase (breaks sucrose, the majority nectar component, into glucose and fructose), amylase (hydrolyses the starches that contaminate the nectar), and the glucose oxidase (converts glucose into gluconic acid and peroxide, both of which are responsible for honey’s antiseptic properties) [2]. The process of reducing the moisture of the nectar begins right after its ingestion by the honeybees (called foragers), before being passed to the worker bees. Afterwards, water loss occurs in parallel with the enzymatic process due to the nectar droplets’ repeated ingestion–regurgitation process and is completed after depositing the product in hexagonal cells in the honeycomb, wherein the bees create an airflow with their wings to evaporate even more moisture until a percentage of 20% is reached. The next step is to seal the honey-containing cells with a wax cap, which ensures the safe storage of the honey [2,3].

There are over 320 different types of honey, and the composition of this natural product can vary substantially depending on the variety of plants from which the nectar is obtained, in addition to the environmental conditions in which the plants grow [4,5].

The European Union contributes to 13% of the world’s production of honey after China (27%). Among European honey producers, during 2020–2021, Romania occupied second place (with 2,353,000 hives) after Spain (with 2,953,000 hives), and Switzerland and Poland (with over 2,000,000 hives) came after Romania. Even with these conditions, the demand for honey on the European market exceeds production, and the European Union is the second largest importer of honey (38.8%), after North America (40.3%), with the main exporter being Asia (mainly China), then Central America and the Caribbean [6].

Honey contains at least 181 chemicals; it is a supersaturated solution of sugars, mainly composed of fructose and glucose along with some oligosaccharides, proteins, vitamins, and minerals [1]. A wide range of minor constituents, such as phenolic acids, flavonoids [7,8], certain enzymes (glucose oxidase, catalase) [9], and amino acids (with proline as their major contributor) [10,11], are also present in honey, and many of them are known to have antioxidant properties.

Fructose and glucose are responsible for honey’s sweetness and viscosity, but the ratio of fructose to glucose can affect its taste and consistency. Minerals such as calcium, iron, and potassium, as well as enzymes, can contribute to the flavor, color, and aroma of honey. The amount and type of amino acids in honey can vary depending on the type of flowers from which the bees collect the nectar, but they have a relatively small effect on the overall properties of honey compared with the other components. The composition of honey changes over time because during storage, the Maillard reaction and/or caramelization can occur when the concentration of monosaccharides decreases and the levels of organic acids, 5-hydroxymethylfurfural (HMF), and furosine increase [12,13].

Honey is a high-caloric product (330 kcal 100 g^−1^); this is especially due to its high sugar content, and its rapid absorption after consumption [14].

Honey presents anti-bacterial and anti-inflammatory properties, and it is used in the treatment of different diseases, such as skin wounds and gastrointestinal issues [1,14,15]. This is due to honey’s high osmotic pressure, acidity, and hydrogen peroxide content [16,17]. Hydrogen peroxide, produced enzymatically, is also responsible for honey’s antibacterial activity. The most important honey enzymes are diastase (amylase), which breaks down starch or glycogen into smaller sugar units, invertase (sucrase, α-glucosidase), which breaks down sucrose into fructose and glucose, and glucose oxidase, which forms hydrogen peroxide and gluconic acid from glucose [18] and acts as a natural preservative and antibacterial agent in honey. Catalase is another enzyme that helps break down the hydrogen peroxide produced by glucose oxidase into water and oxygen, thus preventing the accumulation of harmful amounts of hydrogen peroxide in honey [19].

Monofloral honey, being the most appreciated form of honey, is the main target of adulteration. The quality, aroma, and physicochemical properties of honey within the same floral source vary due to seasonal climatic variations or the geographical location of the apiary [19,20]. Therefore, an important issue in beekeeping concerns the identification of pure honey and the verification of its authenticity.

Honey authenticity consists of two major aspects: the origin of the honey, which includes the botanical origin and geographical provenance, and the way in which the honey was produced, which is related to the harvesting and processing of the honey [21,22]. For botanical origin determination, a melissopalynogical analysis based on the microscopic identification of the pollen type must be performed [23], and for the characterization of the different types of honey, sensory analyses and determinations of some physicochemical characteristics are necessary. Finally, all these results must be correlated for a proper determination of the honey’s origin. Physicochemical parameters of honey types are generally similar, and as such, it is difficult to differentiate between different types of honey based on the physicochemical analysis of this beekeeping product. Under these conditions, ATR-FTIR spectroscopy stood out because it is a non-destructive, fast, reliable, easy, and inexpensive method which enables the authentication of the honey samples. Both near-infrared (NIR) and mid-infrared (MIR) regions of the electromagnetic spectrum have been used in earlier studies for the detection of the botanical origin and geographical provenance of honey samples; the accuracy of classification of the honey samples was greater than 90% [24,25,26].

It is known that any infrared spectrum offers a unique fingerprint; therefore, it is possible to identify a specific compound from all others. The carbohydrate profile of honey revealed that all honey samples possessed a reduced quantity of sugars (mainly fructose and glucose), and small quantities of disaccharides and trisaccharides. The vibrational spectra recorded using ATR-FTIR spectroscopy was found to be a good methodology with which to evaluate sugars in honey [27,28,29]. The sweet and delicious flavor of honey is largely dependent on the volatile and semi-volatile organic compounds present in the honey, the characteristic peaks of these chemicals from IR spectra were used in order to determine the flavor variation in terms of floral origins and honey processes [30,31]. Thus, infrared spectroscopy can be used for the detection of the possible adulteration of honey due to the unique fingerprints of authentic foods in the IR region [32]. The chemometric method (PCA), coupled with ATR-FTIR spectra, allows differentiations between the honey samples [22,24,25,26,29].

Adulterated honey is produced by dissolving sugar and agave, corn, or maple syrup in water or by feeding bees with sugar and syrup, which produces artificial honey [33]. For this reason, knowing the origin and quality of honey is very important. Different food authorities control food validity at national and international levels using codes such as the Codex Alimentarius and European Community standards [34,35].

This study sought to analyse the electrical conductivity (EC), pH, free acidity (FA), moisture, ash, 5-hydroxymethylfurfural (HMF), total sugar content (TSC), and some representatives of the phenolics of the honey produced by bee populations of the *Apis mellifera Carpatica* race in the authorized apiaries of Southern Romania from five botanical sources (sunflower, linden, rapeseed, multifloral, and acacia). The effect of the botanical and geographical origin on the physiochemical properties of honey was evaluated using IR spectroscopy coupled with chemometric analysis.

## 2. Materials and Methods

### 2.1. Chemicals and Reagents

All chemicals and reagents were purchased from Merck, Darmstadt, Germany.

### 2.2. Honey Samples

During 2021–2022, twenty-four samples of Romanian honey were analyzed, with the following geographical origins (GO): *sunflower* (S) from Arges—Costesti and Arges—Gliganu (*AG-C*, and *AG-G*), *linden* (L) from Giurgiu—Bolintin and Tulcea—Topolog (*GR-B*, and *TL-T*), *rapeseed* (R) from Teleorman—Branceni, Arges—Costesti, and Arges—Gliganu (*TR-B*, *AG-C*, and *AG-G*), *multifloral* (M) from Tulcea—Casimcea and Arges—Mozaceni (*TL-C*, and *AG-MZ*), and *acacia* (A) from Arges—Costesti, Arges—Mosoaia, and Arges—Vedea (*AG-C*, *AG-MO*, and *AG-V*). The twenty-four honey samples were produced by bee populations of the *Apis mellifera Carpatica* race in the small or medium-sized apiaries of Southern Romania. All samples were collected directly from the primary producers, without any thermal treatment (based on the fact that raw honey is allowed for marketing in Romania). Immediately after harvesting, the samples were subjected to physicochemical, biochemical, and spectral analysis.

### 2.3. Botanical Origin Identification

The botanical origin (BO) of the Romanian honey samples was confirmed using melissopalynological analysis, in accordance with the methodology suggested by Louveaux et al. (1978) [36]. Both the qualitative results (pollen spectrum of the honey sample) and quantitative results (number of pollen grains per gram of honey) were registered. The pollen spectrum of each honey sample was determined by counting at least 800 pollen grains. Only the pollen grain types with frequencies higher than 1% were considered. For each honey sample, the relative frequency classes were determined in accordance with the international melissopalynological nomenclature using the terms: ‘dominant pollen’ (more than 45% of pollen grains counted), ‘accompanying pollen’ (representing 15–45%), ‘important minor pollen’ (3–15%), and ‘minor pollen’ (less than 3%) [36].

### 2.4. Physicochemical Determinations

The physicochemical parameters were determined in accordance with the Harmonised Methods of the International Honey Commission (2009) [34].

All samples were prepared in triplicate.

*Moisture* and *ash* (mineral content) were determined gravimetrically via oven drying at 105–110 °C, and the calcination of dry residue was determined at 550–600 °C, until the samples were brought to a constant mass. The results were expressed as a percentage (%) of the moisture and ash content.

*Electrical conductivity* (EC) was determined by measuring the electrical resistance of aqueous solutions of honey consisting of 20% dry matter, with a multimeter C-561, at 20 °C. The results were expressed as micro Siemens per centimeters (μS cm^−1^).

*pH* was measured using aqueous honey solutions, consisting of 10% dry matter, using a multimeter Consort C-561.

*Free acidity* (FA) was determined by titrating aqueous solutions of honey consisting of 10% dry matter, using 0.1 M sodium hydroxide solution, until it reached pH 8.30. The results were expressed in milliequivalents of acids per kg of honey (mEq kg^−1^).

*Total sugars content* (TSC) expressed as g glucose (Glu) 100 g^−1^ was determined colorimetrically by following the methodology suggested by Dubois et al. (1956) [37].

*5-hydroxymethylfurfural* (HMF) was determined in aqueous solutions of honey consisting of 20% dry matter using the measurement obtained from the absorbances of the filtered solutions, after clarification with Carrez I and II solutions, at 284 and 336 nm (White method [38]). The results were expressed in milligrams HMF per kg of honey (mg HMF kg^−1^).

### 2.5. Bioactive Compound Determinations

*Total polyphenols content* (TPC) was determined using the methodology suggested by Ciucu-Paraschiv and Hoza (2021) [39]. Honey solutions consisting of 40% dry matter in absolute ethanol were used. The results were expressed as a mg gallic acid equivalent (GAE) 100 g^−1^.

*Total tannin content* (TTC) was determined using the methodology suggested by Giura et al. (2019) [40], using aqueous solutions of honey consisting of 40% dry matter. The results were expressed as a mg gallic acid equivalent (GAE) 100 g^−1^.

*Total flavonoid content* (TFC) was determined using the methodology suggested by Tudor-Radu et al. (2016) [41], using honey solutions consisting of 40% dry matter in absolute ethanol and the results were expressed as a mg catechin equivalent (CE) 100 g^−1^.

*Total antioxidant activity* was determined using the methodology suggested by Lazar et al. (2020) [42], using ethanolic solutions of honey consisting of 40% dry matter. The results were expressed as a percentage of the inhibition of 2,2-diphenyl-1-picrylhydrazyl (DPPH I%).

### 2.6. UV-Vis and ATR-FTIR Analysis

The spectral measurements were made with a UV-Vis Perkin-Elmer Lambda25 and an FTIR Jasco 6300 spectrometer.

An ATR accessory equipped with a diamond crystal (Pike Technologies, Madison, Wisconsin, USA) allows the collection of FTIR spectra directly on a sample without any special preparation. The FTIR spectra were recorded in the region of 4000–400 cm^−1^, with a TGS detector, and apodization Cosine. The spectral data were processed with JASCO Spectra Manager software, version 2. Samples were scanned at a 4 cm^−1^ resolution, accumulation: 100 scans. Background reference spectra were recorded using air after every sample to minimize the interference due to carbon dioxide and water vapor in the atmosphere. Between measurements, the ATR crystal was carefully cleaned using pure acetone (Sigma-Aldrich Co., Saint Louis, MO, USA), then, it was dried with soft tissue [43,44,45].

All measurements were taken at room temperature (T = 23 °C). For each sample, three replicate spectra were recorded to ensure spectral reproducibility and to assess analytical precision; then, the average spectrum was complete.

### 2.7. Chemometric Analysis

Infrared Spectra were exported from Spectra Manager, in an ASCII (dx) format, into the Unscrambler Software (Edition X 10.4, Camo Oslo, Norway) for chemometric analysis. Spectra were preprocessed using the second-derivative transformation, the Savitzky–Golay derivation. The use of spectra derivatives with the Savitzky–Golay algorithm as a chemometric pre-processing technique has been widely reported in most classifications that are based on FTIR spectroscopy [43,44,45]. Multivariate analysis (e.g., principal component analysis, PCA; hierarchical cluster analysis, HCA; linear discriminate analysis, LDA) was previously often used to evaluate and/or classify honey depending on its chemical composition, physicochemical, or biological properties [28,46]. The principal component analysis (PCA) model was developed using cross-validation. PCA was performed on both the entire spectral range (4000 to 400 cm^−1^) and on the MIR ‘fingerprint’ (1700–750 cm^−1^ and 1200–950 cm^−1^). Validation: Cross Validation. Algorithm: Singular Value Decomposition (SDV).

### 2.8. Statistical Analysis

Two-way ANOVA, followed by the Duncan Multiple Range Test at a significance level of α = 0.05 (IBM SPSS 20), were used to study the influence of the botanical origin (BO), year, botanical origin × year interaction, and geographical origin (GO), year, and geographical origin × year interaction on honey quality indicators.

Data were reported as the mean ± standard deviation of at least three replications. Pearson correlation coefficients, r, (at a significance level of 95%) were calculated using IBM SPSS 20 software to measure the strength of the linear relationships between honey quality indicators. Only statistically significant correlations were discussed.

## 3. Results and Discussion

### 3.1. Melissopalynological Analysis

The pollen analysis showed that sunflower honey had the principal pollen *Helianthus annuus* (48.5–74.1%), linden honey had the principal pollen *Tilia tomentosa* (31.8–61.4%), rapeseed honey had the principal pollen *Brassica napus* (46.1–81.7%), and acacia honey had the principal pollen *Robinia pseudoacacia* (24.3–53.1%).

According to Bodó et al. (2021) [47], the minimum percentage of pollen required to classify honey as monofloral is 45%. The authors also noted some exceptions: for *Lamiaceae* origin and thyme honey, the honey requires at least 18% pollen, sage honey requires 20% pollen, as does acacia honey, according to Uršulin-Trstenjak et al. (2017) [48].

In our study, the minimum percentage of pollen was above the mentioned limit, which certifies the monofloral origin of the samples. Based on pollen analysis, the Romanian honey samples were classified in accordance with the botanical origin. The categories of botanical origin were as follows: acacia (six samples), linden (four samples), rapeseed (six samples), sunflower (four samples), and multifloral honey (four samples).

As secondary pollen contributors, *Malus* (apple), *Pyrus* (pear), *Cerasus* (cherry), *Taraxacum* (dandelion), *Antirrhinum* (snapdragon), *Corylus* (hazelnut), and *Salix* (willow) were found for rapeseed honey, and *Brassica*, *Malus* (apple), *Pyrus* (pear), *Cerasus* (cherry), *Taraxacum* (dandelion), *Fragaria* (strawberry), and *Prunus persica* (peach) were found for acacia honey. Linden honey also contains pollen from *Robinia*, *Hypericum* (St. John’s wort), *Rubus* (blackberry, raspberry), *Achilea* (yarrows), and *Sinapis alba* (white mustard), whereas sunflower honey contains pollens from *Tilia* (linden), *Matricaria* (chamomile), *Achilea* (yarrow), and *Silybum* (milk thistle). In multifloral samples, the main pollen was *Brassica* (12.3–37.5%), followed by *Helianthus* (sunflower, *8.0–14%*), *Phacelia* (phacelia heliotrope), *Trifolium* (white/sweet clover), and *Mentha* (mint).

Dobre et al. (2013) [49] found that in multifloral honey, the main species contributing to pollen were *Brassica napus* (dominant) followed by *Bifora radians* (wild bishop), *Tilia* (linden), *Prunus* (plum), *Plantago* (fleaworts), and *Echium* (blueweed). For acacia honey, the authors noted that there was 5–58% *Robinia pseudoacacia* pollen, in rapeseed honey there was 52–93% *Brassica* pollen; however, in multifloral honey, the *Brassica* pollen content was less than 40%. In linden honey, 28.3–88.3% the *Tilia pollen* was reported as being lower than 45%; this was similar to our study. In sunflower honey, the *Helianthus annuus* pollen content was 57.7–65.5% [50]. Uršulin-Trstenjak et al. (2017) [48] found very large oscillations with regard to pollen percentages in acacia honey: between 22% and 71% (average of 43.55%) depending on the harvest area. Halagarda et al. (2020) [51] found that the accompanying pollens (pollen presented in a proportion of 15–45%) in multifloral honey are as follows: rapeseed (38.9–44.6%), white/sweet clover (17.1–39.7%), raspberry (21.8–38.9%), linden (22.7%), phacelia (19.6%), and buckwheat (17.2%). Oroian and Ropciuc (2017) [52] found smaller variation amplitudes and lower maximums for acacia and sunflower honey compared with the present study (45.1–49.6% and 60.1–68.7%, respectively). All these differences (some of them quite large) are mainly due to the effect of climatic factors. The late spring frosts, increased nebulosity, the rains, the strong wind, and also the very high temperatures during the flowering period, cause damage to the floral structures, and thus, they prevent the bees from flying or they simply reduce the accumulation of carbohydrates available to the pollinators [53]. There is no doubt that the spontaneous flora in the area where the beehives were located, which were different from one region to another, left their mark on the results of the melissopalynological analysis.

### 3.2. Botanical Origin Effect on Honey Quality Indicators

The values of some physicochemical parameters, presented in Table 1 (i.e., moisture, ash, electrical conductivity (EC), pH, free acidity (FA), and total sugar content (TSC)) and Table 2 (i.e., hydroxymethylfurfural (HMF), total phenolic (TPC), tannin (TTC), flavonoid content (TFC), and antioxidant activity (DPPH I%)), for the five analyzed types of honey, indicate the influence of the botanical origin (BO), the study year (Year), and the BO × Year interaction.

All five types of tested honey presented a safe moisture content (Table 1) for storage and consumption (12.21–18.74%, data not presented), falling below the maximum value (20%) established by the Codex Alimentarius standard [35]. Moisture can negatively influence the quality of honey when stored as it creates favorable conditions for microbial activity (fermentation, for example). Hence, the presence of a large amount of water must be removed from the honey (to ensure its stability during the storage period) as it affects other quality parameters of the honey. Therefore, high humidity is related to the reduction of thermosensitive compound content (vitamin C, for example); this is because, in order to remove the moisture, the duration of the heat treatment needs to be extended, or higher temperatures are required for dehydration. In addition, as Singh and Singh (2018) [54] mentioned, the heat treatment, as well as the extended storage time, increased the HMF content of honey, especially for honey with a low pH. For this reason, HMF is considered to be a marker of excessive heat treatment, but it is also used to check the adulteration of honey with glucose syrup. On the other hand, Chaikham and Prangthip (2015) [55] reported an increase in TPC and TFC levels in thermally treated honey (at 50 °C and 70 °C), but the treatment did not have a significant effect on antioxidant activity. On the contrary, some studies [56,57] show a significant increase in the antioxidant activity of honey following heat treatments. Even in conditions where honey does not suffer deterioration under dehydration temperatures, the presence of high humidity is correlated with higher costs, as thermal treatment is necessary to bring the honey to a water content that does not affect its stability during storage.

It is appreciated that ash content is an important quality indicator of honey; it reflects the content of mineral elements, and it is also dependent on the botanical origin as it is accepted that an ash content of less than 0.6% indicates the floral origin of the honey [58]. All honey samples analyzed in our study had an ash content (Table 1) lower than 0.6%. Compared with our samples, a larger variation of ash content in honey, from 0.03 to 0.42% (honey from Romania), was reported by Albu et al. (2022) [59]. Regarding Tunisian honey, Boussaid et al. (2018) [60] found ash content in the range of 0.05–0.49% (honey from other European countries) or with maximum limits up to 0.69%. Similarly, in the study by Albu et al. (2021) [61], the highest ash content was determined to exist in linden honey (higher than that reported in the present study), an average level was reported by the authors for multifloral honey, and the minimum level was found in acacia honey.

Electrical conductivity (EC) is another parameter used for honey quality control and to certify its botanical origin and purity [62]. EC is correlated with organic acid content, mineral salts, proteins, and the honey’s color; a lighter color indicates lower conductivity and a darker color indicates higher conductivity [62,63,64]. In this study, EC (Table 1) ranged from 169 to 615 µS cm^−1^ (data not presented), and according to Directive 2014/63/EU [65], the analyzed samples come from nectar honey (origin certified with EC values lower than 0.8 mS cm^−1^ and ash values below 0.6%). Moreover, except for sunflower honey, in 2021, the average EC was lower than 500 µS cm^−1^, a value which, according to Pauliuc et al. (2020) [63], is considered the maximum limit that is reached, with some exceptions, such as EC in the case of pure floral honey. The authors also mention the fact that EC values between 500 and 800 µS cm^−1^ are attributed to mixed honey. Interestingly, the multifloral honey presented EC values lower than 500 µS cm^−1^. Higher values, compared with the upper limit of this study, were 0.637 mS cm^−1^ (for Romanian honey) and 0.689 mS cm^−1^ (honey from Bulgaria), whereas the minimums were set at 0.097 and 0.083 mS cm^−1^ [59]. In general, the EC values reported for Romanian linden and sunflower honey were the highest, multifloral honey had average EC, and rapeseed and acacia honey had the lowest values [59,61,66]. Nevertheless, it must be noted that there were some exceptions with regard to monofloral honey, that had even higher EC values than the 0.8 mS cm^−1^ limit mentioned by some authors. These include: *P. aviculare* (Knot weed), *Gossypium* sp. (cotton honey), *Paliurus spina-christi* (Jerusalem thorn) [67], and *Persea americana* (avocado honey) [68].

The pH values recorded for the analyzed honey samples (Table 1) were within the standard limits (pH 3.40–6.10) that ensure the honey’s freshness, and they were in accordance with the Codex Alimentations (2001) [35]. Unlike pH, free acidity (FA) showed very high variations (Table 1). Rapeseed honey had the lowest FA (5.25 mEq kg^−1^), followed by the group consisting of linden and acacia honey (7.13 and 7.50 mEq kg^−1^, respectively), whereas sunflower honey showed high FA (16.67 mEq kg^−1^). The level of free acidity in the range of 4.00 to 25.00 mEq kg^−1^ (below the maximum allowed of 50 mEq kg^−1^ [34]) indicated the freshness of all samples and the absence of any fermentation processes in the tested honey. Honey generally has a slightly acidic character due to the content of organic acids (predominantly gluconic acid); this is also an indicator of the botanical and geographical origin of the honey and it contributes to the appearance (color) and taste of the honey [69]. Highly acidic honey is the result of sugar fermentation, which is responsible for both the honey’s taste and its microbiological stability. Moreover, it is also positively correlated with the honey’s mineral content [70]. High pH values (which differ depending on the studied year) for linden honey, and medium values for acacia honey, were determined by Albu et al. [59,61], whereas for rapeseed, acacia, and multifloral honey, lower pH values were found in the Czech Republic and Poland [71]. Free acidity is considered to be an indicator that the honey can be subjected to long storage times or ineffective heat treatments; this is because fermentation is a process that increases the honey’s natural FA. Moreover, according to some authors, FA increased slightly in honey that was stored, especially after the first 20 months [69]. FA is related to honey content in tartaric, citric, oxalic, acetic, and other organic acids, but it also depends on nectar or bee secretions [64].

Multifloral honey showed the highest total sugar content (TSC), 69.64 g Glu 100 g^−1^, followed by acacia (63.79 g Glu 100 g^−1^), whereas the lowest level of sugars was recorded for linden honey (58.05 g Glu 100 g^−1^). Rapeseed and sunflower honey had a similar sugar content to both acacia and linden honey (62.00 and 60.82 g Glu 100 g^−1^, respectively). TSC over 60% (except for linden honey, with 58.05 gGlu 100 g^−1^) showed characteristics of nectar-derived honey. The TSC content determined for multifloral honey in this study fell below the values reported by Abdulkhaliq and Swaileh (2017) [72]. Moreover, higher sugar contents were also determined for acacia honey [73,74], which were similar to that of rapeseed honey [74]. However, TSC in multifloral honey fell within the limits found by Nešović et al. (2020) [75]. It is generally considered that nectar-derived honey has a minimum level of 60% sugar (as a sum of fructose and glucose), whereas honeydew-derived honey has a smaller minimum sugar content of 45% [35]. Regarding the *Tilia* species, Jacquemart et al. (2018) [76] mentioned that nectar is the main source of carbohydrates for flower-visiting bees, and they correlated observations of mortality among bees visiting *Tilia* flowers with data from the literature regarding the presence of toxic carbohydrates (mannose), of some alkaloids (mainly nicotine), and the low carbohydrate content of these flowers (the authors state that the bees die of hunger). This latter observation could justify the low level of total sugar in the analyzed linden honey samples, which could remove the uncertainty regarding its floral origin. Jacquemart et al. (2018) [76] analyzed the sugar content of the nectar of some linden species and found that TSC decreased in the order *T. tomentosa*, *T. platyphillos*, *T × europaea*, *T. cordata*. In Romania, the first species to bloom is *T. platyphyllos*; after 10–15 days, the downy lime *T. cordata* blooms, and after 21–22 days, *T. tomentosa* blooms. In total, this equates to a period of approximately 30 days (from June to July), though it varies from one locality to another and from one year to another, depending on pedoclimatic conditions [71]. The nectar secretion of linden flowers begins at temperatures of at least 16 °C, they increase visibly after 20 °C, and they stop completely over 33 °C. Therefore, during the flowering period, drought, strong and cold winds, heavy rains that produce large amounts of water and have long duration periods (June is often a rainy month in Romania), low temperatures, and fogs cause damage to flowers and reduce or stop their nectar secretions [71]. Low TSC in linden honey could be explained as a consequence of both the lack of sugars in linden nectar and the rainy season in June. Juan-Borrás et al. (2014) [77] compared linden honey from Romania with linden honey from the Czech Republic and found that the highest amount of glucose plus fructose was in honey from the Czech Republic (75% sum of glucose plus fructose). Linden honey from Romania contained a higher total sugar content (71% sum of glucose plus fructose) than was found in the present study. In the same study, the sum of the two sugars decreased in sunflower honey (76.6%), linden honey (73.9%), and acacia honey (73.7%), and was substantially higher than those found in our study. This aspect could be justified by the different climatic conditions during the years in which the honey was produced and the honey’s geographical origins in the two studies.

5-hydroxymethylfurfural (HMF) is an intermediate that is formed during the Maillard reaction, wherein heat treatment is applied to acidic honey, and the honey is subjected to conditions of prolonged storage. In general, the presence of HMF is associated with a drop in honey quality. As previously discussed, treating honey with heat does not always negatively influence its quality, and some products with antioxidant activity are also formed in the Maillard reaction [63]. However, in the case of the Maillard reaction, we can also speak of a reduction in the nutritional quality of honey, as some of the essential amino acids are destroyed [69]. Regarding the limits of HMF concentrations in honey, some authors cite the absence of HMF in fresh honey [78], though a maximum level of 40 mg HMF kg^−1^ is allowed according to European legislation, and an even higher level (by 60 mg kg^−1^) is allowed according to Brazilian legislation. Moreover, as Codex Alimentarius (2001) [35] claims, for some tropical-origin honey, HMF should not exceed 80 mg kg^−1^. The HMF content of all tested honey (Table 2) fell within the International Honey Commission Standards’ [34] standard recommended limit (the maximum concentration of 40 mg kg^−1^), and it was confirmed that the tested honey was not subjected to any heat treatment. The highest HMF content was found in linden honey (33.94 mg kg^−1^). Compared with our study, most of the data reported in the literature indicated lower levels (sometimes even tenfold) of HMF [66,79,80,81,82], whereas linden honey was also mentioned by Matović et al. (2018) [81] due to its high HMF level (17.86 mg kg^−1^). Nevertheless, Guerzou et al. (2021) [83] found higher HMF content levels, at 90.7 and 117.7 mg kg^−1^, and they cited authors that explained that the high HMF content was as a result of the heat treatment (responsible for an increase of up to 145.5 mg kg^−1^) and inadequate storage conditions.

The antioxidant activity of honey is mainly due to phenolic compounds, the main sources of which are pollen and nectar [84]. The secondary metabolism products of plants are synthesized under abiotic and biotic stress conditions. Another role that these phenolic compounds have is that of attractants for pollinators, which are also consumed by the bees, along with the nectar, and they are later transferred into the honey. Among the compounds with antioxidant activity analyzed in the study, the total phenolic content (TPC) showed a variation that was lower compared with those reported by Albu et al. (2022) [59] (1.00–142.61 mg GAE 100 g^−1^) for Romanian honey, and Al-Mamary et al. (2002) [85] (56.32–246.21 mg GAE 100 g^−1^) for Yemeni honey; however, it was higher compared with the results reported by Tomczyk et al. (2019) [71] (20–47 mg GAE 100 g^−1^) for Polish and Slovakian honey, and similar to the results reported by Nešović et al. (2020) [75] for Montenegro honey (39.16–110.65 mg GAE 100 g^−1^). Contrary to this study, acacia honey from Malaysia had a higher level of phenolic compounds (196.50 mg GAE 100 g^−1^) [86], as did multifloral honey from Serbia (139 mg GAE 100 g^−1^) [64] and Montenegro (70.02 mg GAE 100 g^−1^) [75]. Moreover, TPC decreased in the same order (sunflower, multifloral, and rapeseed honey) in the study of Pauliuc et al. (2020) [63], but the values determined by these authors were at least five times lower.

Al-Farsi et al. (2018) [87] mention that the main phenolic compounds present in honey are flavonoids and some phenolic acids, which are compounds that influence its taste and appearance (color in particular). In a previous study, Hamdy et al. (2009) [88] stated that the flavonoids in honey mainly come from nectar and pollen, but also propolis. Another, even earlier study by Tomás-Barberán et al. (1993) [89] showed that the ratio between propolis-derived flavonoids and pollen-nectar-derived flavonoids could be correlated with the geographical origin of the honey. The authors hypothesized that flavonoids derived from propolis are found in higher proportions in European honey (Spain and Italy), and in the temperate zones of the northern hemisphere (where poplar predominates), than in honey from other regions. Unlike our results, Kaškonienė et al. (2009) [90] determined that linden honey has a flavonoid content of 32.0 µg rutin equivalents (RE) g^−1^, and almost twice the lower level of flavonoids in rapeseed honey (13.5 µg RE g^−1^). Moreover, unlike the present study (in which TFC = 12.81% of TPC, only slightly higher compared with TTC), Sabatier et al. (1992) [91] stated that in monofloral honey, flavonoids comprise the majority of phenolic compounds (up to 42%). Data presented in the literature regarding TFC vary greatly. Thus, Al-Farsi et al. (2018) [87] found that in an acacia species of honey (*Acacia tortilis*) levels of flavonoids were 2143 mg kg^−1^ (1613–2890 mg kg^−1^), and TPC varied around the value of 2236 mg kg^−1^ (1624–2898 mg kg^−1^). In the same study, the TFC and TPC levels found in multifloral honey were 925 mg kg^−1^ (521–1354 mg kg^−1^) and 1066 mg kg^−1^ (842–1384 mg kg^−1^), respectively.

Compared with this study, the analysis of four types of honey from the Czech Republic [71] indicated a reduction of DPPH I% in the following order: linden, multifloral, acacia, rapeseed honey, and higher values of this indicator. A similar ranking, but lower values compared with the discussed study, was obtained by the same authors for the same types of honey that were collected from Poland [71].

Except for antioxidant activity (DPPH), all analyzed parameters were significantly influenced by the botanical origin of honey (*p* ˂ 0.001).

The results presented in Table 1 and Table 2 showed that sunflower honey had the highest moisture (15.53%), ash (0.21%), electrical conductivity (483.92 µS cm^−1^), free acidity (16.67 mEq kg^−1^), phenolics (167.59 mg GAE 100 g^−1^), tannins (69.05 mg GAE 100 g^−1^), flavonoids (19.00 mg CE 100 g^−1^), and antioxidant activity (28.16%), whereas multifloral honey presented the highest total sugar content (69.64 g Glu 100 g^−1^).

A significant effect of the year (*p* ˂ 0.001) on honey moisture content was noted in 2022, where higher moisture was found in the honey samples (15.16%) compared with 2021 (13.65%) (Table 1). A significant effect of the botanical origin × year interaction (BO × year) (*p* ˂ 0.001) was highlighted by the differences in humidity recorded for sunflower and linden honey in 2021 and 2022 (Table S1), but this was not observed in rapeseed, multifloral, and acacia honey (*p* > 0.05). In addition, although in the first year of the study, there were no significant oscillations in terms of humidity among the five types of honey, they were noted in 2022.

Table 1 showed no differences between the ash contents of the average samples collected over the two years, and no significant effect of the botanical origin × year interaction (*p* > 0.05) was observed. However, it was noted that there was no common trend in terms of increasing or decreasing ash content between 2021 and 2022, and the only significant oscillations in ash content were determined for linden and multifloral honey (Table S1). A similar effect concerning the botanical origin (BO) in 2021 and 2022 was observed (Table 1); rapeseed honey had the lowest ash content and sunflower honey had the highest ash content.

Although EC did not vary significantly over the studied years (*p* = 0.075), a significant effect of the botanical origin × year interaction (*p* = 0.007) was highlighted; the EC significantly decreased for acacia and rapeseed honey between 2021 and 2022 (Table 1). No differences regarding the effect of the botanical origin, during either of the studied years, were observed.

Significant differences (*p* = 0.015) were recorded between the average pH levels recorded in 2021 (4.38) and 2022 (4.49). In addition, a significant effect of the botanical origin × year of study interaction was also highlighted. Related to this effect, it was observed that the strong differences between the honey pH levels recorded in 2021 (the values were arranged in four groups in accordance with homogeneity) were mitigated the following year (when only two homogeneous subsets were displayed) (Table 1). Except for linden honey (pH decreased significantly between 2021 and 2022), for all other botanical origins, the honey pH levels increased between 2021 and 2022, with a significant increase for sunflower and acacia honey only (Table S1).

On average, the most acidic honey was collected in 2021 (9.82 mEq kg^−1^), compared with 7.50 mEq kg^−1^ in 2022 (*p* ˂ 0.001, Table 1). The differences between the years, depending on the honey’s botanical origin, were particularly apparent upon observation of the increase of FA in linden honey (from low, in 2021, to medium acidity, in 2022), and the reduction of FA in acacia honey (from medium, in 2021, to low acidity, in 2022 (Table S1).

Furthermore, on average, the sugar content (TSC) of honey collected in the second year of the study was greater than in 2021 (Table 1), and except for acacia honey, this tendency was observed across all other four types of honey (Table S1). In addition, among the five types of honey, a significant variation in TSC between 2021 and 2022 was found only for linden honey. Similar variations in both FA and TSC were noted in 2021 and 2022; FA had the highest level in sunflower honey and the lowest level in rapeseed honey, whereas the highest TSC level was found in multifloral honey, and the minimum level was found in linden honey (Table 1).

Regarding the effect of the year, a higher HMF level was observed in 2021 (23.36 mg kg^−1^) compared with 2022 (20.07 mg kg^−1^) (Table 2). As shown in Table S2, except for sunflower honey, significant differences in HMF content were registered between 2021 and 2022.

During the two study years, TPC decreased from 129.48 mg GAE 100 g^−1^ in 2021 to 98.65 mg GAE 100 g^−1^ in 2022 (Table 2). Along with this reduction, the differences between honey samples were accentuated; in 2022, the honey types, based on TPC content, were rigidly segregated into four groups, starting with sunflower honey, followed by multifloral honey, rapeseed honey, linden honey, and finally, acacia honey. The most stable TPC content between 2021 and 2022 was found in rapeseed, linden, and multifloral honey.

Honey tannins (TTC) represented about 43.18% of the total phenolic content (data not presented). A more intense variation in TTC compared with TPC (Table 2) was found, mainly due to the effect of the year (*p* ˂ 0.001), but also the honey’s botanical origin (*p* ˂ 0.001) and botanical origin × year interaction (*p* = 0.001). The highest TTC was observed in sunflower and acacia honey (69.05 and 62.73 mg GAE 100 g^−1^, respectively). The average tannin content was found in multifloral honey (45.15 mg GAE 100 g^−1^), and the lowest TTC in linden honey (28.72 mg GAE 100 g^−1^). No significant differences were recorded between the tannin contents of rapeseed honey (39.00 mg GAE 100 g^−1^) and multifloral or linden honey. On average, TTC decreased in 2022 compared with 2021, similarly to TPC. The most intense reductions were recorded for sunflower honey (by 60.60 mg GAE 100 g^−1^) and multifloral honey (by 42.85 mg GAE 100 g^−1^), whereas the smallest difference was observed for rapeseed honey (12.49 mg GAE 100 g^−1^), although all were statistically assured (*p* ˂ 0.05, Table S2).

The total flavonoid content (TFC) represented approximately 12.81% of the TPC of the honey analyzed in the study (data not presented). Given the significant effect of the botanical origin (*p* ˂ 0.001, Table 2), TFC showed a maximum level in sunflower honey (19.00 mg CE 100 g^−1^), followed by multifloral honey (17.39 mg CE 100 g^−1^), rapeseed honey (13.74 mg CE 100 g^−1^), linden honey (12.93 mg CE 100 g^−1^), and acacia honey (11.83 mg CE 100 g^−1^). Similarly to TPC, linden honey did not differ significantly compared with rapeseed or acacia honey in terms of TFC. The level of flavonoids decreased non-significantly from 2021 to 2022 (15.02 and 14.20 mg CE 100 g^−1^, respectively). Nevertheless, the most intense reduction was observed for sunflower honey (5.81 mg CE 100 g^−1^). In addition, an exception was registered in the case of multifloral honey, where the TFC increased in 2022 by 3.08 mg CE 100 g^−1^. Nevertheless, linden honey, followed by rapeseed, and acacia honey, appeared to have the most stable flavonoid content.

Unlike other honey quality indicators, antioxidant activity (DPPH I%) was influenced only by the botanical origin of honey (*p* = 0.001, Table 2). Two groups of values were observed: a group with high DPPH I% (24.95–28.16%), in which sunflower, linden, rapeseed, and multifloral honey were found, and a class with low antioxidant activity, in which only acacia honey (19.57%) was included. Similarly to TFC, rapeseed honey presented with an unchanged level of antioxidant activity, as did the linden and acacia samples. Botanical origin had a slightly stronger effect on DPPH I% in 2022 (25.82%) compared with 2021 (24.10%), but it was non-significant.

### 3.3. Geographical Origin Effect on Honey Quality Indicators

Significant variations in honey quality indicators were also observed between samples collected from different regions, and these differences varied depending on the year in which the honey was produced (Tables S3–S6). Some exceptions were still observed. For example, the moisture content of the rapeseed, multifloral, and acacia honey samples varied depending on the collection area, as well as the year of the study (as previously noted). The lack of GO × year interaction was due to the similar evolution of moisture in all samples (Tables S3 and S5), regardless of geographical origin (in all cases the moisture contents determined in 2022 were higher than in 2021 for rapeseed and acacia, whereas for multifloral honey, the differences between 2021 and 2020 were insignificant both in TL-C and AG-AZ). In addition, for sunflower honey, the variations between the collection areas and between years in terms of ash content were insignificant (Tables S3 and S5). However, the evolution of the ash content over the years was different in AG-C (ash increased in 2022 by 0.10%) compared with AG-G (ash decreased in 2022 by 0.9%). The ash content of linden honey did not significantly depend on the collection area; a decrease in ash content in 2022 was evident both in TL-T and in GR-B. The ash content of rapeseed honey varied, but insignificantly, between 2021 and 2022, and for multifloral honey, no combined GO × year effect was registered, neither in the case of ash nor in the case of FA.

Among the compounds with antioxidant activity (Tables S4 and S6), the only deviation observed was that of flavonoids in linden honey, with insignificant variations found across the years of the study.

The indicators that showed constant evolution, with higher levels in 2022 in all regions, were moisture, HMF (with the small exception of sunflower honey from AG-G), TPC (with the two exceptions of rapeseed honey from TR-B and multifloral honey from TL-C), and TTC.

Differences between honey collected from different regions have been frequently reported in the literature [71,92,93,94], and the differences observed in cases where the honey had the same botanical origin were attributed to the climatic conditions of the collection areas [92]. Tomczyk et al. (2019) [71] found that linden honey from the two regions (Poland and Slovakia) differed the most in terms of antioxidant and physicochemical parameters, and in both countries, rapeseed honey exhibited the most similar properties. Some authors even managed to classify the types of honey according to their geographical origin, with the help of a chemometric model [25,46,73,80].

Mădaş et al. (2020) [94] stated that it is difficult to find adequate markers with which to establish the origin of honey, especially because these markers vary greatly with the botanical origin of the honey. For this reason, more sensitive methods are recommended for the identification of honey, such as chromatographic techniques; for instance, HPLC or GC-MS, but also Infrared and Raman spectroscopy. In any case, for the development of an accurate method with which to identify geographical origin, it is necessary to utilize very large databases.

### 3.4. Correlation Matrix between Quality Indicators of Honey

In the analysis of the correlations between the quality indicators, it is necessary to take into account the fact that the analyzed honey samples were neither subjected to thermal treatment nor were they stored for a long time. The correlation matrix (Table 3) indicates that moisture correlated negatively with TTC (r = −0.352 **) and positively with DPPH I% (r = 0.273 *). Likewise, other correlations established by DPPH I% were positive ones, such as ash (r = 0.280 *), HMF (r = 0.237 *), and TFC (r = 0.292 *), though it correlated negatively with TTC (r = −0.286 *). A high level of ash was correlated with high EC (r = 0.689 **), FA (r = 0.470 **), HMF (r = 0.285 *), TPC (r = 0.464 **), TTC (r = 0.265 *), TFC (r = 0.405 **), and DPPH I% (r = 0.280 *). Except for DPPH I%, the same types of correlations were established in the case of EC. Honey with a low pH showed a high level of sugar (r = −0.307 **), TPC (r = −0.536 **), TTC (r = −0.511 **), and TFC (r = −0.593 **), and sugar was positively correlated with TFC (r = 0.289 *). Finally, positive correlations were observed between TPC, TTC, and TFC (r = 0.738 **, for TPC and TTC, r = 0.806 ** for TPC and TFC, r = 0.419 ** for TTC and TFC).

The analysis of the correlations between TTC and moisture content, in accordance with the botanical origin of the honey (data not-presented), indicated that, in all cases, the correlations were negative; this was very significant for linden (r = −0.879 ***), distinctly significant for acacia (r = −0.626 **) and sunflower (r = −0.709 **), and significant for rapeseed (r = −0.534 *). The only case in which the correlation between TTC and moisture was insignificant (although still negative) was multifloral honey (r = −0.188). To an extent, the correlations between TPC and moisture (data not presented) were similar: significantly negative for sunflower (r = −0.601 *) and rapeseed (r = −0.485 *), and distinctly significant for acacia (r = −0.714 **). In this case, for multifloral honey, the TPC correlation with moisture was insignificantly positive (r = 0.246), and for linden, although negative, it was not statistically significant (r = −0.496). Regarding the correlation between TTC, TPC, and moisture, over the two years of study (data not presented), it could be observed that in 2021, the correlations were negative, and in 2022, they were positive, but both were statistically insignificant.

Regarding the correlation between the biochemical quality parameters of honey, similarly significant positive correlations between TFC and DPPH I% in Algerian honey, as well as some Malaysian samples, were also reported by Khalil et al. (2012) [95]. The authors [95] also reported a significant positive correlation between TPC and DPPH I%, but with a lower correlation coefficient compared with TFC (r = 0.615 * for TPC and r = 0.888 ** for TFC). They also found strong correlations between DPPH I% and proline (r = 0.956 **) and ascorbic acid (r = 0.785 **) [95]. In our study, the correlation between antioxidant activity (DPPH I%) and TFC was positive and significant, whereas the correlation between antioxidant activity and TPC, although positive, was not statistically significant. These results indicate that, although found in low concentrations compared with other classes of phenolic compounds, honey′s flavonoids are among the main contributors to its antioxidant activity.

The antioxidant activity of honey is due to components such as flavonoids, phenolic acids, enzymes, and vitamins, but also minerals, such as copper and iron [86]. Some authors identified 54 mineral elements in honey, classified into major, minor, and heavy metals [96]. In our study, honey with high moisture contents also presented a high ash content and high antioxidant activity. This relationship between humidity and antioxidant activity could be justified by the ash content. It is known that honey contains several mineral elements, but the most important, from the point of view of antioxidant activity, are manganese (cofactor of enzymes with an antioxidant role), copper (involved in the synthesis of superoxide dismutase), zinc (involved in the production of antioxidants and synthesis of superoxide dismutase), selenium (involved in the synthesis of glutathione peroxidase) and iron (with a role in the neutralization of active radicals) [97].

### 3.5. ATR-FTIR and Chemometric Analysis

**Figure 1:**
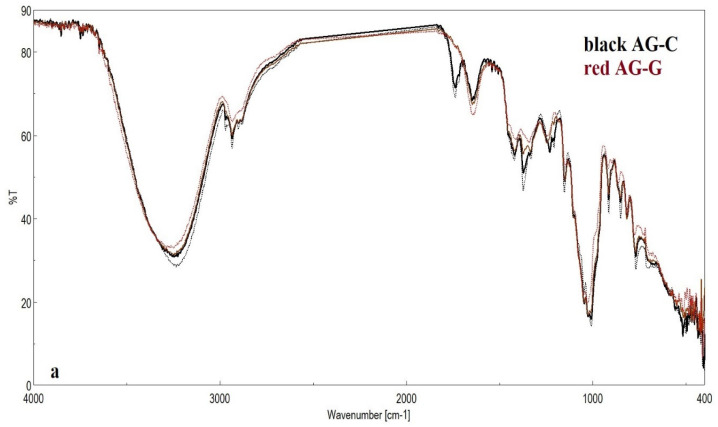

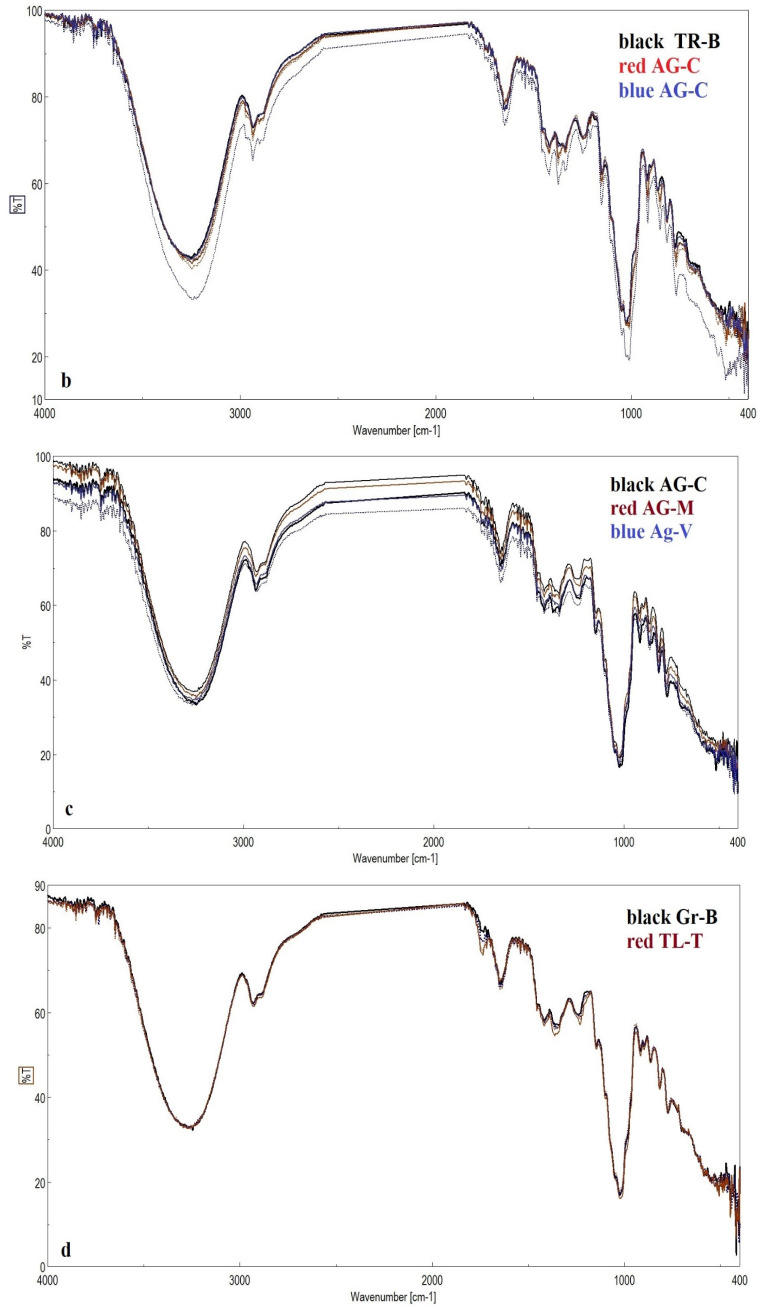

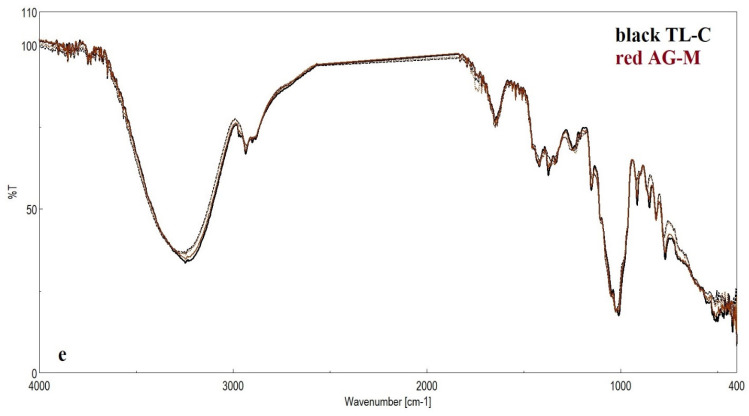
ATR-FTIR spectra of honey samples with different botanical origins: sunflower (**a**), rapeseed (**b**), acacia (**c**), linden (**d**), and multifloral (**e**) (line continuous—2021, dotted line—2022).

To compare the honey samples, FTIR spectroscopy was used as an efficient method. Figure 1 shows the ATR-FTIR spectra of the tested honey with major high bands. The characteristic differences between the FTIR spectral analyses for honey samples were observed. Five major areas were identified in the MIR domain, and the fingerprint region was localized between 3600–900 cm^−1^. **Area 1** (3350–3600 cm^−1^) was assigned to the stretching vibrations of OH (from water, alcohols, phenols, and carbohydrates). **Area 2** (2800–2900 cm^−1^) corresponds with the C-H stretching vibrations of CH_3_ and CH_2_ from lipids and lipid derivatives. **Area 3** is complex (1500–1760 cm^−1^), and corresponds with bending vibrations C=O, C-N stretching (acids, amide I), and amide II absorption (primarily N-H bending coupled with a C-N stretching vibrational mode). **Area 4** (1500–1230 cm^−1^) corresponds with stretching C-O, deformation C-H, and deformation N-H, whereas **Area 5** (1230–915 cm^−1^) is assigned to C-O stretching in carbohydrates and the phosphate band (and 965 cm^−1^ to fructose [98]). The representative ATR-FTIR spectrum of sunflower honey with the described regions is presented in Figure S1.

Table 4 shows the exact position of the bands, together with the assignment of relevant vibrations, in specific functional groups. In carboxylic acids and alcohols, the O–H stretching vibration band is quite wide, measuring in the range of 3300–2500 cm^−1^, with the largest band measuring at 3000 cm^−1^ [99]; this is the same area as the stretching vibration region for carbon and aromatic C–H groups [100]. The peaks around 2930 cm^−1^ are characteristic of C–H stretching in carboxylic acids and NH_3_ stretching in free amino acids [99,101]. The absorption band measuring at around 1640 cm^−1^ is due to both water and a small amount of protein molecules [22,100]. The peaks measuring from 1175 to 940 cm^−1^ corresponded with C–O stretching in carbohydrates, as follows: 1148 cm^−1^ was specific to sucrose; 1087 cm^−1^ and 1043 cm^−1^ indicated the presence of glucose and fructose; and 983 cm^−1^ and 965 cm^−1^ indicated the presence of fructose [22,29,98]. In the analysed Romanian honey samples, the stretching vibration band of the C=O carboxylic acids group measured between 1760–1690 cm^−1^, although the exact position of the band depended on whether the acid was saturated or unsaturated, dimerized or associated, and so on [86].

**Figure 2:**
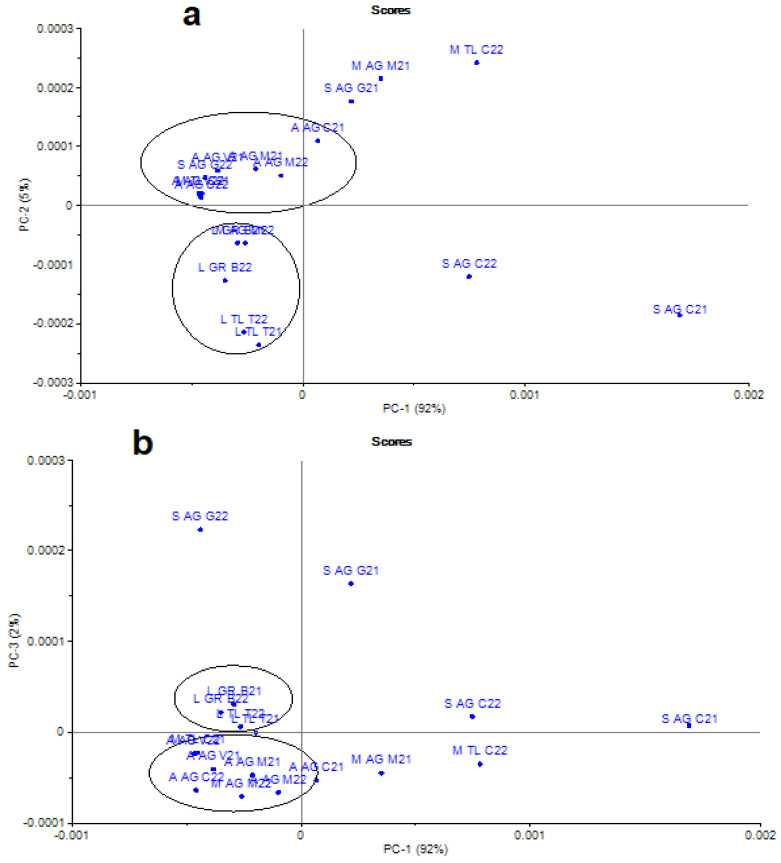
Two-dimensional scores obtained from the PCA of FTIR spectra of honey for the first two PCs (**a**), and PC3 versus PC1 (**b**).

There were no significant differences observed between the MIR spectra of the analyzed honey samples. Nevertheless, using chemometric analysis, honey sample discrimination was possible. For the selected regions, 1700–750 cm^−1^ and 1200–950 cm^−1^, a good result when discriminating between multifloral honey and acacia honey was found. For the considered honey samples, the first three principal components (PCs) represented 99% of the total variance (PC1 = 92%, PC2 = 5%, and PC3 = 2%). This indicates that these three components were sufficient to provide a good separation between the groups (Figure 2). This region includes the region at 1150–1000 cm^−1^, which was frequently preferred for the spectral analysis of carbohydrates during IR spectroscopy. Linden honey was separated from acacia honey, multifloral honey, and sunflower honey, respectively.

## 4. Conclusions

The best-represented pollen was from *Brassica napus* in rapeseed honey (46.1–81.7%), followed by *Helianthus annus* in sunflower honey (48.5–74.1%), *Tillia tomentosa* in linden honey (31.8–61.4%), and *Brassica* pollen was best represented in multifloral honey (12.3–37.5%).

The honey’s botanical origin represented the main variability source. Sunflower honey stood out given its multiple qualities (ash, free acidity, antioxidant compounds, and antioxidant activity). It was followed by multifloral honey, with its maximum sugar level, an appreciable content in terms of antioxidant compounds, and antioxidant activity, and linden honey, with its lower of sugar and organic acid content, but high antioxidant activity. Based on the quality parameter variations between 2021 and 2022, the least stable honey was linden honey, followed by sunflower and acacia honey, whereas rapeseed honey was the opposite. Among the phenolic compounds, flavonoids are the most strongly positively correlated with the antioxidant activity of honey, and they are found in higher concentrations in honey with lower pH levels, but high sugar content.

The chemometric method, coupled with ATR-FTIR spectra, revealed a clear separation between linden honey acacia, multifloral, and sunflower honey.

The presented results revealed a series of correlations between compounds (such as the relationship between phenolic compounds and moisture content), which, to be correctly understood and explained, require further study.

## Figures and Tables

**Table 1:** Honey moisture, ash, electrical conductivity (EC), pH, free acidity (FA), and total sugar content (TSC) influenced by botanical origin (BO), study year (Year), and BO × Year interaction (means ± SD are presented).

		Moisture (%)	Ash (%)	EC (µS cm^−1^)	pH	FA (mEq kg^−1^)	TSC (g Glu 100 g^−1^)
BO	S	15.53 ± 0.28 a	0.21 ± 0.01 a	483.92 ± 14.80 a	3.95 ± 0.05 c	16.67 ± 0.50 a	60.82 ± 1.84 bc
	L	14.62 ± 0.28 b	0.20 ± 0.01 a	437.00 ± 14.80 b	4.75 ± 0.05 a	7.13 ± 0.50 c	58.05 ± 4.18 c
	R	14.41 ± 0.23 b	0.09 ± 0.01 c	210.44± 12.09 d	4.64 ± 0.04 a	5.25 ± 0.40 d	62.00 ± 5.46 bc
	M	13.15 ± 0.28 c	0.14 ± 0.01 b	374.25± 14.80 c	4.34 ± 0.05 b	9.04 ± 0.50 b	69.64 ± 11.30 a
	A	14.36 ± 0.23 b	0.14 ± 0.01 b	243.22± 12.09 d	4.41 ± 0.04 b	7.50 ± 0.40 c	63.79 ± 4.27 b
	*p*	*˂0.001*	*˂0.001*	*˂0.001*	*˂0.001*	*˂0.001*	*˂0.001*
Year	2021	13.65 ± 0.98 b	0.15 ± 0.07 a	343.11 ± 122.60 a	4.38 ± 0.35 b	9.82 ± 5.75 a	61.24± 6.13 b
	2022	15.16 ± 1.53 a	0.15 ± 0.06 a	315.44 ± 121.44 a	4.49 ± 0.29 a	7.50 ± 2.68 b	64.49± 7.28 a
	*p*	*˂0.001*	*0.752*	*0.075*	*0.015*	*˂0.001*	*0.014*
BO × Year	*p*	***˂0.001***	***0.051***	***0.007***	***0.012***	***˂0.001***	***0.332***
BO-Year (2021) BO-Year (2022)	S	13.85 ± 0.39 a	0.20 ± 0.10 a	523.00 ± 94.71 a	3.88 ± 0.02 d	21.00 ± 3.41 a	60.15 ± 0.63 bc
	L	13.25 ± 0.72 a	0.23 ± 0.04 a	418.83 ± 85.29 b	4.84 ± 0.15 a	6.17 ± 1.51 c	54.62 ± 3.15 c
	R	13.97 ± 1.10 a	0.09 ± 0.02 c	231.33 ± 29.42 c	4.62 ± 0.27 b	5.44 ± 1.47 c	59.69 ± 7.08 bc
	M	12.93 ± 0.52 a	0.12 ± 0.03 b	354.67 ± 53.69 b	4.22 ± 0.13 c	10.08 ± 2.48 b	66.73 ± 3.33 a
	A	13.95 ± 1.29 a	0.15 ± 0.03 b	276.78 ± 72.53 c	4.28 ± 0.09 c	9.00 ± 2.02 b	64.28 ± 5.60 ab
	*p*	*0.176*	*˂0.001*	*˂0.001*	*˂0.001*	*˂0.001*	0.002
	S	17.22 ± 1.26 a	0.21 ± 0.06 a	444.83 ± 17.70 a	4.02 ± 0.06 b	12.33 ± 1.03 a	61.49± 2.44 b
	L	15.98 ± 0.90 b	0.16 ± 0.03 ab	455.17 ± 5.95 a	4.66 ± 0.03 a	8.08 ± 0.58 b	61.48± 0.56 b
	R	14.84 ± 0.71 c	0.09 ± 0.04 c	189.56 ± 19.94 c	4.67 ± 0.23 a	5.06 ± 0.39 c	64.31± 1.09 b
	M	13.37 ± 0.67 d	0.17 ± 0.03 ab	393.83 ± 29.42 b	4.46 ± 0.34 a	8.00 ± 2.21 b	72.56 ± 15.79 a
	A	14.76 ± 1.29 c	0.13 ± 0.06 bc	209.67 ± 33.79 c	4.54 ± 0.03 a	6.00 ± 0.00 c	63.30± 2.63 b
	*p*	*˂0.001*	0.001	*˂0.001*	*˂0.001*	*˂0.001*	0.035

BO = sunflower, linden, rapeseed, multifloral, and acacia; year = 2021 and 2022; BO-year = each group of five botanical origins (sunflower, linden, rapeseed, multifloral, and acacia) collected in 2021 and 2022, analyzed separately. For each BO mean, at least 12 determinations are presented (12 determinations for S, L, and M, and 18 determinations for R and A). For each year, means of at least 12 determinations were presented (2 for S, L, and M and 3 for R and A). For each BO-Year, means of at least 6 determinations were presented (6 for S, L, and M, and 9 for R and A). Means with the same letter in each column are not significantly different at a 5% level, in accordance with Duncan′s Multiple Range Test. *p* values for the significance of the BO and Year influence were presented, and calculated in accordance with the One-Way Analysis of Variance (at a significance level of α = 0.05). *p* values for the significance of the BO × Year influence were calculated in accordance with the Two-Way Analysis of Variance (at a significance level of α = 0.05). S = sunflower honey, L = linden honey, R = rapeseed honey, M = multifloral honey, A = acacia honey.

**Table 2:** Honey hydroxymethylfurfural (HMF), total phenolic (TPC), tannin (TTC), flavonoid content (TFC), and antioxidant activity (DPPH I%) influenced by botanical origin (BO), study year (Year), and BO × year interaction (means ± SD are presented).

		HMF (mg kg^−1^)	TPC (mg GAE 100 g^−1^)	TTC (mg GAE 100 g^−1^)	TFC (mg CE 100 g^−1^)	DPPH I%
BO	S	19.37 ± 0.49 c	167.59 ± 8.05 a	69.05± 4.43 a	19.00 ± 0.54 a	28.16 ± 1.71 a
	L	33.94 ± 0.49 a	102.39 ± 8.05 c	28.72± 4.43 c	12.93 ± 0.54 cd	28.14 ± 1.71 a
	R	18.28 ± 0.40 c	102.53 ± 6.57 c	39.00± 3.62 bc	13.74 ± 0.44 c	26.12 ± 1.40 a
	M	25.44 ± 0.49 b	125.33 ± 8.05 b	45.15± 4.43 b	17.39 ± 0.54 b	24.95 ± 1.71 a
	A	16.07 ± 0.40 d	90.18 ± 6.57 c	62.73± 3.62 a	11.83 ± 0.44 d	19.57 ± 1.40 b
	*p*	*˂0.001*	*˂0.001*	*˂0.001*	*˂0.001*	*0.001*
Year	2021	23.36 ± 7.13 a	129.48 ± 53.24 a	65.43 ± 28.43 a	15.02 ± 3.88 a	24.10 ± 6.98 a
	2022	20.07 ± 6.10 b	98.65 ± 19.46 b	33.08 ± 13.34 b	14.20 ± 3.04 a	25.82 ± 6.62 a
	*p*	*˂0.001*	*˂0.001*	*˂0.001*	*0.067*	*0.178*
BO × Year	*p*	***˂0.001***	***0.003***	***0.001***	***˂0.001***	***0.112***
BO-Year (2021) BO-Year (2022)	S	19.80 ± 0.33 c	211.10 ± 70.28 a	99.35 ± 31.53 a	21.90 ± 4.22 a	24.47 ± 2.93 ab
	L	35.53 ± 1.49 a	110.84 ± 12.86 b	40.02 ± 3.52 d	12.97 ± 0.17 c	30.43 ± 6.28 a
	R	19.91 ± 3.27 c	103.24 ± 4.46 b	45.25 ± 5.02 cd	13.99 ± 0.98 bc	26.03 ± 10.00 ab
	M	29.38 ± 2.22 b	139.30 ± 32.47 b	66.57 ± 8.50 bc	15.85 ± 2.49 b	22.12 ± 1.29 b
	A	17.04 ± 1.05 d	107.20 ± 43.23 b	79.18 ± 28.59 ab	12.28 ± 1.03 c	19.03 ± 3.99 b
	*p*	*˂0.001*	*˂0.001*	*˂0.001*	*˂0.001*	*0.018*
	S	18.93 ± 0.95 c	124.09 ± 2.63 a	38.75 ± 1.47 ab	16.09 ± 1.39 b	31.85 ± 1.93 a
	L	32.35 ± 0.77 a	93.94 ± 3.34 c	17.42 ± 6.69 d	12.89 ± 0.51 cd	25.85 ± 0.58 ab
	R	16.66 ± 1.92 d	101.83 ± 12.12 c	32.76 ± 4.99 bc	13.48 ± 0.80 c	26.21 ± 8.15 ab
	M	21.50 ± 1.17 b	111.37 ± 0.79 b	23.72 ± 2.24 cd	18.93 ± 3.35 a	27.78 ± 2.09 a
	A	15.09 ± 0.88 e	73.16 ± 11.49 d	46.28 ± 15.85 a	11.38 ± 1.10 d	20.10 ± 7.27 b
	*p*	*˂0.001*	*˂0.001*	*˂0.001*	*˂0.001*	*0.008*

BO = sunflower, linden, rapeseed, multifloral, and acacia; year = 2021 and 2022; BO-year = each group of honey from five botanical origins (sunflower, linden, rapeseed, multifloral, and acacia), which were collected in 2021 and 2022, were analyzed separately. For each BO mean, at least 12 determinations are presented (12 determinations for S, L, and M, and 18 determinations for R and A). For each year, means of at least 12 determinations were presented (2 for S, L, and M, and 3 for R and A). For each BO-Year mean, at least 6 determinations were presented (6 for S, L, and M, and 9 for R and A). Means with the same letter in each column are not significantly different at a 5% level, in accordance with Duncan′s Multiple Range Test. *p* values for the significance of the BO and Year influence were presented and calculated in accordance with the One-Way Analysis of Variance (at a significance level of α = 0.05). *p* values for the significance of the BO × Year influence were calculated in accordance with the Two-Way Analysis of Variance (at a significance level of α = 0.05). S = sunflower honey, L = linden honey, R = rapeseed honey, M = multifloral honey, A = acacia honey.

**Table 3:** Correlation matrix between honey moisture, ash, electrical conductivity (EC), pH, free acidity (FA), total sugar content (TSC), HMF, total phenolic (TPC), tannin (TTC), flavonoid (TFC) content, and antioxidant activity, expressed as DPPH radical inhibition activity of honey (r values are presented).

		Ash	EC	pH	FA	TSC	HMF	TPC	TTC	TFC	DPPH I%
Moisture	Pearson correlation	0.120 ***	0.040	−0.064	0.021	−0.052	−0.212	−0.226	−0.352 **	−0.133	0.273 *
	Sig. (2-tailed)	0.315	0.740	0.593	0.864	0.667	0.074	0.056	0.002	0.266	0.020
Ash	Pearson correlation	1	0.689 **	−0.242 *	0.470 **	−0.033	0.285 *	0.464 **	0.265 *	0.405 **	0.280 *
	Sig. (2-tailed)		˂0.001	0.041	˂0.001	0.782	0.015	˂0.001	0.025	˂0.001	0.017
EC	Pearson correlation		1	−0.406 **	0.731 **	−0.115	0.466 **	0.690 **	0.282 *	0.621 **	0.167
	Sig. (2-tailed)			˂0.001	˂0.001	0.335	˂0.001	˂0.001	0.017	˂0.001	0.161
pH	Pearson correlation			1	−0.798 **	−0.307 **	0.212	−0.536 **	−0.511 **	−0.593 **	0.004
	Sig. (2-tailed)				˂0.001	0.009	0.073	˂0.001	˂0.001	˂0.001	0.973
FA	Pearson correlation				1	0.049	−0.023	0.846 **	0.658 **	0.776 **	0.041
	Sig. (2-tailed)					0.685	0.849	˂0.001	˂0.001	˂0.001	0.734
TSC	Pearson correlation					1	−0.125	−0.005	−0.008	0.289 *	−0.128
	Sig. (2-tailed)						0.295	0.970	0.950	0.014	0.283
HMF	Pearson correlation						1	0.063	−0.217	0.012	0.237 *
	Sig. (2-tailed)							0.601	0.066	0.922	0.045
TPC	Pearson correlation							1	0.738 **	0.806 **	0.013
	Sig. (2-tailed)								˂0.001	˂0.001	0.917
TTC	Pearson correlation								1	0.419 **	−0.286 *
	Sig. (2-tailed)									˂0.001	0.015
TFC	Pearson correlation									1	0.292 *
	Sig. (2-tailed)										0.013

* Significant at *p* ˂ 0.05, ** Significant at *p* ˂ 0.01, *** Significant at *p* ˂ 0.001.

**Table 4:** The location of the maxima of absorption bands FTIR in the tested honey samples.

Honey	Sunflower (S)				Rapeseed (R)						Acacia (A)						Linden (L)				Multifloral (M)			
Year	2021	2022	2021	2022	2021	2022	2021	2022	2021	2022	2021	2022	2021	2022	2021	2022	2021	2022	2021	2022	2021	2022	2021	2022
Harvest zone	AG-C		AG-G		TR-B		AG-C		AG-G		AG-C		AG-MO		AG-V		GR-B		TL-T		TL-C		AG-MZ	
ν(O-H) in H_2_O	3246	3235	3246	3248	3246	3246	3244	3247	3248	3245	3246	3260	3246	3243	3244	3269	3245	3251	3260	3268	3245	3244	3245	3245
ν(C-H) tretching of carboxylic acids + ν(NH_3_) of free aminoacids	2936	2935	2935	2933	2933	2935	2935	2935	2933	2935	2935	2930	2935	2933	2931	2935	2929	2927	2927	2927	2935	2930	2935	2933
CH_3_ sym stretch	2899	2881	2885	2887	2887	2883	2881	2880	2887	2885	2883	2888	2880	2882	2892	2892	2881	2888	2883	2884	2879	2883	2883	2879
δ(O-H) from H_2_O	1646	1646	1645	1644	1646	1646	1646	1646	1646	1646	1646	1646	1646	1646	1646	1646	1646	1646	1646	1646	1646	1646	1646	1646
δ(O-H) in C-OH group + δ(C-H) in the alkenes	1419	1420	1419	1415	1418	1419	1418	1419	1418	1419	1418	1418	1418	1418	1418	1418	1418	1417	1417	1418	1419	1418	1419	1416
Stretching C-O, deformation C-H, deformation N-H	1372	1373	1372	1373	1373	1373	1373	1373	1366	1373	1373	1357	1367	1365	1362	1360	1363	1364	1363	1362	1373	1373	1373	1362
ν(C–H) + ν(C–O) in carbohydrates	1231 1151	1231 1152	1247 1150	1244 1148	1245 1147	1247 1150	1246 1150	1247 1151	1246 1149	1247 1151	1233 1148	1243 1145	1233 1148	1234 1148	1243 1147	1242 1145	1243 1147	1232 1146	1230 1146	1231 1146	1247 1151	1243 1146	1247 1150	1232 1146
ν(C-O) in C-OH group + ν(C-C) in carbohydrates	1046 1009	1046 1008	1046 1024	1049 1028	1046 1026	1047 1010	1046 1011	1046 1010	1046 1025	1046 1010	1051 1024	1051 1024	1052 1024	1050 1024	1048 1024	1049 1025	1051 1024	1049 1024	1051 1023	1049 1025	1046 1009	1046 1024	1047 1010	1048 1024
δ(C–H)	915	914	915	917	917	915	915	914	916	914	916	917	916	915	917	916	916	917	916	917	915	917	915	916

## Data Availability

The data presented in this study are available on request from the corresponding author. The data are not publicly available due to privacy.

## Supplementary Materials

The following supporting information can be downloaded at: https://www.mdpi.com/article/10.3390/foods12112134/s1, Table S1: Honey moisture, ash, electrical conductivity (EC), pH, free acidity (FA), and total sugar content (TSC) influenced by the study year depending on honey botanical origin (BO); Table S2: Honey hydroxymethylfurfural (HMF), total phenolic (TPC), tannin (TTC), flavonoid content (TFC), and antioxidant activity (DPPH I%) influenced by the study year depending on the honey’s botanical origin (BO); Table S3: Honey moisture, ash, electrical conductivity (EC), pH, free acidity (FA), and total sugar content (TSC) influenced by geographical origin (GO), year, and GO × year interaction; Table S4: Honey hydroxymethylfurfural (HMF), total phenolic (TPC), tannin (TTC), flavonoid content (TFC), and antioxidant activity (DPPH I%) influenced by geographical origin (GO), year and GO × year interaction for each botanical origin (BO); Table S5: Honey moisture, ash, electrical conductivity (EC), pH, free acidity (FA), and total sugar content (TSC) influenced by the study year depending on the geographical origin (GO) for each botanical origin (BO) of honey; Table S6: Honey hydroxymethylfurfural (HMF), total phenolic (TPC), tannin (TTC), flavonoid content (TFC), and antioxidant activity (DPPH I%) influenced by the study year depending on geographical origin (GO) for each BO of honey; Figure S1: Representative ATR-FTIR spectrum of sunflower honey (1—stretching vibrations of OH, 2—C-H stretching vibrations of CH3 and CH2 from lipids, 3 and 4—C=O, amide I, amide I, C-N from proteins, 5—C-O stretching in carbohydrates, phosphate band).
