# Supplementary material for: Botanical Origin Influence on Some Honey Physicochemical Characteristics and Antioxidant Properties
Source: Foods. 2023 May 25;12(11):2134. doi: 10.3390/foods12112134 (PMC10253141; doi:10.3390/foods12112134)
Supplement: Supplementary file 1 [file foods-12-02134-s001.zip › foods-2394186-supplementary.pdf]

Table S1. Honey moisture, ash, electrical conductivity (EC), pH, free acidity (FA), and total sugar content (TSC) influenced by the study year depending on honey botanical origin (BO).

		Moisture (%)	Ash (%)	Electrical conductivity (mS/cm)	pH	Free acidity (mEq/kg)	Total sugar content (g Glu/100 g)
S	2021	13.85±0.39 b	0.20±0.10 a	523.00±94.71 a	3.88±0.02 b	21.00±3.41 a	60.15±0.63 a
	2022	17.22±1.26 a	0.21±0.06 a	444.83±17.70 a	4.02±0.06 a	12.33±1.03 b	61.49±2.44 a
	P	*<0.001*	*0.819*	*0.075*	*<0.001*	*<0.001*	*0.224*
L	2021	13.25±0.72 b	0.23±0.04 a	418.83±85.29 a	4.84±0.15 a	6.17±1.51 b	54.62±3.15 b
	2022	15.98±0.90 a	0.16±0.03 b	455.17±5.95 a	4.66±0.03 b	8.08±0.58 a	61.48±0.56 a
	P	*<0.001*	*0.008*	*0.322*	*0.017*	*0.016*	*<0.001*
R	2021	13.97±1.10 a	0.09±0.02 a	231.33±29.42 a	4.62±0.27 a	5.44±1.47 a	59.69±7.08 a
	2022	14.84±0.71 a	0.09±0.04 a	189.56±19.94 b	4.67±0.23 a	5.06±0.39 a	64.31±1.09 a
	P	*0.065*	*0.720*	*0.003*	*0.677*	*0.453*	*0.071*
M	2021	12.93±0.52 a	0.12±0.03 b	354.67±53.69 a	4.22±0.13 b	10.08±2.48 a	66.73±3.33 a
	2022	13.37±0.67 a	0.17±0.03 a	393.83±29.42 a	4.46±0.34 a	8.00±2.21 a	72.56±15.79 a
	P	*0.231*	*0.018*	*0.148*	*0.133*	*0.156*	*0.397*
A	2021	13.95±1.29 a	0.15±0.03 a	276.78±72.53 a	4.28±0.09 b	9.00±2.02 a	64.28±5.60 a
	2022	14.76±1.29 a	0.13±0.06 a	209.67±33.79 b	4.54±0.03 a	6.00±0.00 b	63.30±2.63 a
	P	*0.200*	*0.444*	*0.023*	*<0.001*	*<0.001*	*0.644*

Mean±SD from all geographical origins is presented.
Means with the same letter in each column are not significantly different at a 5% level, in accordance with Duncan`s Multiple Range Test.
P = Year influence significance = presented in accordance with the One-Way Analysis of Variance (at a significance level of α=.05).
S= sunflower honey, L= linden honey, R= rapeseed honey, M= multifloral honey, A= acacia honey.

Table S2. Honey hydroxymethylfurfural (HMF), total phenolic (TPC), tannin (TTC), flavonoid content (TFC), and antioxidant activity (DPPH I%) influenced by the study year depending on the honey's botanical origin (BO).

BO	Year	HMF (mg/kg)	TPC (mg GAE/100 g)	TTC (mg GAE/100 g)	TFC (mg CE/100 g)	DPPH I%
S	2021	19.80 ±0.33 a	211.10±70.28 a	99.35±31.53 a	21.90±4.22 a	24.47±2.93 b
	2022	18.93±0.95 a	124.09±2.63 b	38.75±1.47 b	16.09±1.39 b	31.85±1.93 a
	P	*0.062*	*0.013*	*0.001*	*0.010*	*<0.001*
L	2021	35.53±1.49 A	110.84±12.86 A	40.02±3.52 A	12.97±0.17 A	30.43±6.28 A
	2022	32.35±0.77 B	93.94±3.34 B	17.42±6.69 B	12.89±0.51 A	25.85±0.58 A
	P	*0.001*	*0.011*	*<0.001*	*0.730*	*0.106*
R	2021	19.91±3.27 a	103.24±4.46 a	45.25±5.02 a	13.99±0.98 a	26.03±10.00 a
	2022	16.66±1.92 b	101.83±12.12 a	32.76±4.99 b	13.48±0.80 a	26.21±8.15 a

	P	*0.020*	*0.748*	*<0.001*	*0.243*	*0.968*
M	2021	29.38±2.22 A	139.30±32.47 A	66.57±8.50 A	15.85±2.49 A	22.12±1.29 B
	2022	21.50±1.17 B	111.37±0.79 A	23.72±2.24 B	18.93±3.35 A	27.78±2.09 A
	P	*<0.001*	*0.061*	*<0.001*	*0.101*	*<0.001*
A	2021	17.04±1.05 a	107.20±43.23 a	79.18±28.59 a	12.28±1.03 a	19.03±3.99 a
	2022	15.09±0.88 b	73.16±11.49 b	46.28±15.85 b	11.38±1.10 a	20.10±7.27 a
	P	*0.001*	*0.036*	*0.008*	*0.093*	*0.703*

Mean±SD from all geographical origins is presented.
Means with the same letter in each column are not significantly different at a 5% level, in accordance with Duncan`s Multiple Range Test.
P = Year influence significance presented in accordance with the One-Way Analysis of Variance (at a significance level of *α*=.05).
S= sunflower honey, L= linden honey, R= rapeseed honey, M= multifloral honey, A= acacia honey.

Table S3. Honey moisture, ash, electrical conductivity (EC), pH, free acidity (FA), and total sugar content (TSC) influenced by geographical origin (GO), year, and GO × year interaction.

BO	GO-Year	Moisture (%)	Ash (%)	EC (µS/cm)	pH	FA (mEq/kg)	TSC (g Glu/100 g)
S	AG-C	14.92±0.13 a	0.18±0.01 a	448.67±1.15 a	3.93±0.01 a	15.50±0.19 a	59.99±0.03 a
	AG-G	16.14±0.13 a	0.23±0.01 a	519.17±1.15 a	3.97±0.01 a	17.83±0.19 a	61.65±0.03 a
	2021	13.85±0.39 B	0.20±0.10 A	523.00±94.71 A	3.88±0.02 B	21.00±3.41 A	60.15±0.63 B
	2022	17.22±1.26 A	0.21±0.06 A	444.83±17.70 B	4.02±0.06 A	12.33±1.03 B	61.49±2.44 A
P	*GO*	*0.001*	*0.177*	*<0.001*	*<0.001*	*0.002*	*<0.001*
	Year	*<0.001*	*0.766*	*<0.001*	*<0.001*	*0.000*	*<0.001*
	GO ×Year	*0.002*	*0.029*	*<0.001*	*<0.001*	*<0.001*	*<0.001*
L	TL-T	15.29±0.13 a	0.21±0.01 a	473.33±1.15 a	4.68±0.01 a	8.00±0.19 a	59.74±0.03 a
	GR-B	13.95±0.13 a	0.19±0.01 a	400.67±1.15 b	4.82±0.01 a	6.25±0.19 b	56.36±0.03 a
	2021	13.25±0.72 B	0.23±0.04 A	418.83±85.29 B	4.84±0.15 A	6.17±1.51 B	54.62±3.15 B
	2022	15.98±0.90 A	0.16±0.03 B	455.17± 5.95 A	4.66±0.03 B	8.08±0.58 A	61.48±0.56 A

P	*GO*	*<0.001*	*0.435*	*<0.001*	*<0.001*	*<0.001*	*<0.001*
	Year	*<0.001*	*0.015*	*<0.001*	*<0.001*	*<0.001*	*<0.001*
	GO × Year	*<0.001*	*0.671*	*<0.001*	*<0.001*	*0.005*	*<0.001*
R	AG-C	13.62±0.13 b	0.07±0.01 b	188.33±1.15 b	4.55±0.01 b	4.75±0.19 b	66.61±0.03 a
	TR-B	14.09±0.13 b	0.12±0.01 a	234.33±1.15 a	4.49±0.01 b	6.25±0.19 a	60.80±0.03 b
	AG-G	15.51±0.13 a	0.08±0.01 b	208.67±1.15 ab	4.89±0.01 a	4.75±0.19 b	58.59±0.03 b
	2021	13.97±1.10 B	0.09±0.02 A	231.33±29.42 A	4.62±0.27 B	5.44±1.47 A	59.69±7.08 B
	2022	14.84±0.71 A	0.09±0.04 A	189.56±19.94 B	4.67±0.23 A	5.06±0.39 B	64.31±1.09 A
P	*GO*	*<0.001*	*0.004*	*<0.001*	*<0.001*	*<0.001*	*<0.001*
	Year	*<0.001*	*0.553*	*<0.001*	*<0.001*	*0.014*	*<0.001*
	GO ×Year	*0.212*	*0.009*	*<0.001*	*<0.001*	*<0.001*	*<0.001*
M	TL-C	12.66±0.13 b	0.12±0.01 a	363.17±1.15 a	4.55±0.01 a	6.92±0.19 b	60.92± 0.03 b
	AG-MZ	13.65±0.13 a	0.16±0.01 a	385.33±1.15 a	4.12±0.01 b	11.17±0.19 a	78.36± 0.03 a
	2021	12.93±0.52 B	0.12±0.03 A	354.67±53.69 B	4.22±0.13 B	10.08±2.48 A	66.73± 3.33 B
	2022	13.37±0.67 A	0.17±0.03 B	393.83±29.42 A	4.46±0.34 A	8.00±2.21 B	72.56±15.79 A
P	*GO*	*<0.001*	*0.034*	*<0.001*	*<0.001*	*<0.001*	*<0.001*
	Year	*0.022*	*0.005*	*<0.001*	*<0.001*	*<0.001*	*<0.001*
	GO × Year	*0.496*	*0.083*	*<0.001*	*<0.001*	*0.217*	*<0.001*
A	AG-C	12.90±0.13 c	0.11±0.01 b	283.83±1.15 a	4.45±0.01 a	8.83±0.19 a	62.55±0.03 b
	AG-V	15.85±0.13 a	0.13±0.01 ab	206.83±1.15 a	4.45±0.01 a	6.83±0.19 a	60.35±0.03 b
	AG-MO	14.32±0.13 b	0.17±0.01 a	239.00±1.15 a	4.33±0.01 a	6.83±0.19 a	68.46±0.03 a
	2021	13.95±1.29 B	0.15±0.03 A	276.78±72.53 B	4.28±0.09 B	9.00±2.02 A	64.28±5.60 A
	2022	14.76±1.29 A	0.13±0.06 A	209.67±33.79 A	4.54±0.03 A	6.00±0.00 B	63.30±2.63 B
P	*GO*	*<0.001*	*<0.001*	*<0.001*	*<0.001*	*<0.001*	*<0.001*
	Year	*<0.001*	*0.053*	*<0.001*	*<0.001*	*<0.001*	*<0.001*
	GO × Year	*0.336*	*<0.001*	*<0.001*	*<0.001*	*<0.001*	*<0.001*

Mean±SD from all geographical origins is presented. Means with the same letter in each column are not significantly different at a 5% level, in accordance with Duncan`s Multiple Range Test.
P values for GO and Year influence significance were presented in accordance with the One-Way Analysis of Variance (at a significance level of α=.05). *P* values for the significance of the GO x Year influence were calculated in accordance with the Two-Way Analysis of Variance (at a significance level of α=.05).
S= sunflower honey, L= linden honey, R= rapeseed honey, M= multifloral honey, A= acacia honey.
AG-C=Arges-Costesti, AG-G=Arges-Gliganu, AG-MO=Arges-Mosoaia, AG-MZ=Arges-Mozaceni, AG-V=Arges-Vedea, GR-B=Giurgiu-Bolintin, TR-B=Teleorman-Branceni, TL-C=Tulcea-Casimcea, TL-T=Tulcea-Topolog.

Table S4. Honey hydroxymethylfurfural (HMF), total phenolic (TPC), tannin (TTC), flavonoid content (TFC), and antioxidant activity (DPPH I%) influenced by geographical origin (GO), year and GO × year interaction for each botanical origin (BO).

BO	GO-Year	HMF (mg/kg)	TPC (mg GAE/100 g)	TTC (mg GAE/100 g)	TFC (mg EC/100 g)	DPPH (I%)
S	AG-C	19.08±0.09 a	136.71±0.01 a	53.99±0.01 a	17.70±0.05 a	27.71±0.02 a
	AG-G	19.65±0.09 a	198.47±0.01 a	84.11±0.01 a	20.30±0.07 a	28.62±0.02 a
	2021	19.80±0.33 A	211.10±70.28 A	99.35±31.53 A	21.90±4.22 A	24.47±2.93 B
	2022	18.93±0.95 B	124.09± 2.63 B	38.75± 1.47 B	16.09±1.39 B	31.85±1.93 A
P	*GO*	*<0.001*	*<0.001*	*<0.001*	*<0.001*	*<0.001*
	Year	*<0.001*	*<0.001*	*<0.001*	*<0.001*	*<0.001*
	GO ×Year	*<0.001*	*<0.001*	*<0.001*	*<0.001*	*<0.001*
L	TL-T	33.60±0.09 a	106.73±0.01 a	27.27±0.01 a	12.63±0.05 b	25.01±0.02 b

	GR-B	34.28±0.09 a	98.05±0.01 a	30.17±0.01 a	13.23±0.04 a	31.27±0.02 a
	2021	35.53±1.49 A	110.84±12.86 A	40.02±3.52 A	12.97±0.17 A	30.43±6.28 A
	2022	32.35±0.77 B	93.94± 3.34 B	17.42±6.69 B	12.89±0.51 A	25.85±0.58 B
P	*GO*	*<0.001*	*<0.001*	*<0.001*	*<0.001*	*<0.001*
	Year	*<0.001*	*<0.001*	*<0.001*	*0.243*	*<0.001*
	GO × Year	*<0.001*	*<0.001*	*<0.001*	*0.001*	*<0.001*
R	AG-C	20.02±0.09 a	101.30±0.01 b	37.83±0.01 a	13.59±0.06 a	22.18±0.02 a
	TR-B	19.82±0.09 a	112.43±0.01 a	42.13±0.01 a	13.93±0.05 a	29.55±0.02 a
	AG-G	15.02±0.09 b	93.88±0.01 c	37.06±0.01 a	13.70±0.05 a	26.63±0.02 a
	2021	19.91±3.27 A	103.24± 4.46 A	45.25±5.02 A	13.99±0.98 A	26.03±10.00 B
	2022	16.66±1.92 B	101.83±12.12 B	32.76±4.99 B	13.48±0.80 B	26.21±8.15 A
P	*GO*	*<0.001*	*<0.001*	*<0.001*	*<0.001*	*<0.001*
	Year	*<0.001*	*<0.001*	*<0.001*	*<0.001*	*<0.001*
	GO ×Year	*<0.001*	*<0.001*	*<0.001*	*<0.001*	*<0.001*
M	TL-C	25.92±0.09 a	110.15±0.01 b	40.25±0.01 a	14.73±0.04 b	23.41±0.02 a
	AG-MZ	24.97±0.09 a	140.51±0.01 a	50.05±0.01 a	20.05±0.06 a	26.49±0.02 a
	2021	29.38±2.22 A	139.30±32.47 A	66.57±8.50 A	15.85±2.49 B	22.12±1.29 B
	2022	21.50±1.17 B	111.37±0.79 B	23.72±2.24 B	18.93±3.35 A	27.78±2.09 A
P	*GO*	*<0.001*	*<0.001*	*<0.001*	*<0.001*	*<0.001*
	Year	*<0.001*	*<0.001*	*<0.001*	*<0.001*	*<0.001*
	GO × Year	*<0.001*	*<0.001*	*<0.001*	*<0.001*	*<0.001*
A	AG-C	16.22±0.09 a	118.69±0.01 a	77.62±0.01 a	12.16±0.05 a	13.36±0.02 c
	AG-V	15.82±0.09 a	70.45±0.01 b	48.45±0.01 a	11.18±0.07 a	26.13±0.02 a
	AG-MO	16.17±0.09 a	81.41±0.01 b	62.13±0.01 a	12.16±0.07 a	19.21±0.02 b
	2021	17.04±1.05 A	107.20±43.23 A	79.18±28.59 A	12.28±1.03 A	19.03±3.99 B
	2022	15.09±0.88 B	73.16±11.49 B	46.28±15.85 B	11.38±1.10 B	20.10±7.27 A
P	*GO*	*<0.001*	*<0.001*	*<0.001*	*<0.001*	*<0.001*
	Year	*<0.001*	*<0.001*	*<0.001*	*<0.001*	*<0.001*
	GO × Year	*<0.001*	*<0.001*	*<0.001*	*<0.001*	*<0.001*

Mean±SD from all geographical origins is presented. Means with the same letter in each column are not significantly different at a 5% level, in accordance with Duncan`s Multiple Range Test.
P values for the significance of the GO and Year influence were presented in accordance with the One-Way Analysis of Variance (at a significance level of α=.05). *P* values for the significance of GO x Year influence were calculated in accordance with the Two-Way Analysis of Variance (at a significance level of α=.05).
S= sunflower honey, L= linden honey, R= rapeseed honey, M= multifloral honey, A= acacia honey. AG-C=Arges-Costesti, AG-G=Arges-Gliganu, AG-MO=Arges-Mosoaia, AG-MZ=Arges-Mozaceni, AG-V=Arges-Vedea, GR-B=Giurgiu-Bolintin, TR-B=Teleorman-Branceni, TL-C=Tulcea-Casimcea, TL-T=Tulcea-Topolog.

Table S5. Honey moisture, ash, electrical conductivity (EC), pH, free acidity (FA), and total sugar content (TSC) influenced by the study year depending on the geographical origin (GO) for each botanical origin (BO) of honey.

BO	GO	Year	Moisture (%)	Ash (%)	EC (µS/cm)	pH	FA (mEq/kg)	TSC (g Glu/100 g)
S	AG-C	2021	13.74±0.37 b	0.13±0.03 a	436.67±6.11 b	3.89±0.02 b	18.00±1.00 a	60.72±0.11 a
		2022	16.10±0.27 a	0.23±0.08 a	460.67±5.51 a	3.96±0.01 a	13.00±1.00 b	59.26±0.08 b
		P	*0.001*	*0.107*	*0.007*	*0.003*	*0.004*	*<0.001*
	AG-G	2021	13.95±0.45 b	0.28±0.08 a	609.33±5.13 a	3.86±0.01 b	24.00±1.00 a	59.58±0.06 b
		2022	18.33±0.41 a	0.19±0.03 a	429.00±1.00 b	4.07±0.01 a	11.67±0.58 b	63.72±0.11 a
		P	*<.001*	*0.168*	*<0.001*	*<0.001*	*<0.001*	*<0.001*
L	TL-T	2021	13.83±0.49 b	0.24±0.01 a	496.67±3.06 b	4.70±0.02 a	7.50±0.50 a	57.50±0.06 b

		2022	16.74±0.40 a	0.17±0.04 b	450.00±2.00 a	4.66±0.02 a	8.50±0.50 a	61.98±0.05 a
		P	*0.001*	*0.027*	*<0.001*	*0.060*	*0.070*	*<0.001*
	GR-B	2021	12.67±0.19 b	0.22±0.06 a	341.00±1.00 b	4.97±0.01 a	4.83±0.29 b	51.74±0.07 b
		2022	15.22±0.38 a	0.16±0.01 a	460.33±2.08 a	4.66±0.04 b	7.67±0.29 a	60.97±0.05 a
		P	*<0.001*	*0.196*	*<0.001*	*<0.001*	*<0.001*	*<0.001*
R	AG-C	2021	13.01±0.68 b	0.09±0.02 a	192.33±0.58 a	4.72±0.01 a	4.17±0.29 b	69.06±0.11 a
		2022	14.22±0.30 a	0.05±0.01 b	184.33±0.58 b	4.38±0.01 b	5.33±0.29 a	64.16±0.06 b
		P	*0.047*	*.017*	*<.001*	*<0.001*	*0.008*	*<0.001*
	TR-B	2021	13.62±0.41 b	0.10±0.03 b	254.33±1.53 a	4.27±0.01 b	7.33±0.29 a	55.96±0.05 b
		2022	14.56±0.16 a	0.13±0.01 a	214.33±7.37 b	4.70±0.01 a	5.17±0.29 b	65.64±0.13 a
		P	*0.020*	*0.100*	*0.001*	*<0.001*	*0.001*	*<0.001*
	AG-G	2021	15.29±0.19 b	0.07±0.01 a	247.33±0.58 a	4.85±0.01 b	4.83±0.29 a	54.05±0.07 b
		2022	15.73±0.05 a	0.09±0.02 a	170.00±1.00 b	4.92±0.03 a	4.67±0.29 a	63.13±0.10 a
		P	*0.016*	*0.326*	*<0.001*	*0.013*	*0.519*	*<0.001*
M	TL-C	2021	12.49±0.27 a	0.08±0.00 b	305.67±1.53 b	4.34±0.01 b	7.83±0.29 a	63.69±0.11 a
		2022	12.82±0.31 a	0.16±0.05 a	420.67±1.53 a	4.77±0.01 a	6.00±0.00 b	58.14±0.07 b
		P	*0.233*	*0.043*	*<0.001*	*<0.001*	*<0.001*	*<0.001*
	AG-MZ	2021	13.37±0.10 a	0.15±0.00 b	403.67±0.58 a	4.10±0.01 b	12.33±0.29 a	69.76±0.07 b
		2022	13.93±0.34 a	0.17±0.00 a	367.00±1.00 b	4.15±0.01 a	10.00±0.50 b	86.97±0.06 a
		P	*0.055*	*0.001*	*<0.001*	*0.001*	*0.002*	*<0.001*
A	AG-C	2021	12.45±0.07 b	0.17±0.00 a	373.33±2.08 a	4.34±0.01 b	11.67±0.29 a	64.74±0.09 a
		2022	13.34±0.08 a	0.05±0.00 b	194.33±2.08 b	4.56±0.01 a	6.00±0.00 b	60.37±0.06 b
		P	*<0.001*	*<0.001*	*<0.001*	*<0.001*	*<0.001*	*<0.001*
	AG-V	2021	15.40±0.26 b	0.11±0.00 a	233.00±1.00 a	4.33±0.02 b	7.67±0.29 a	57.60±0.08 b
		2022	16.30±0.18 a	0.15±0.03 a	180.67±0.58 b	4.57±0.01 a	6.00±0.00 b	63.11±0.15 a
		P	*0.008*	*0.105*	*<0.001*	*<0.001*	*0.001*	*<0.001*
	AG-MO	2021	14.00±0.18 b	0.16±0.03 a	224.00±1.00 b	4.16±0.02 b	7.67±0.29 a	70.49±0.08 a
		2022	14.65±0.08 a	0.19±0.02 a	254.00±1.00 a	4.50±0.01 a	6.00±0.00 b	66.43±0.07 b
		P	*0.005*	*0.186*	*<0.001*	*<0.001*	*0.001*	*<0.001*

Mean±SD from all geographical origins is presented. Means with the same letter in each column are not significantly different at a 5% level, in accordance with Duncan`s Multiple Range Test.
P= significance of the year's influence, presented in accordance with the One-Way Analysis of Variance (at a significance level of α=.05).
S= sunflower honey, L= linden honey, R= rapeseed honey, M= multifloral honey, A= acacia honey.
AG-C=Arges-Costesti, AG-G=Arges-Gliganu, AG-MO=Arges-Mosoaia, AG-MZ=Arges-Mozaceni, AG-V=Arges-Vedea, GR-B=Giurgiu-Bolintin, TR-B=Teleorman-Branceni, TL-C=Tulcea-Casimcea, TL-T=Tulcea-Topolog.

Table S6. Honey hydroxymethylfurfural (HMF), total phenolic (TPC), tannin (TTC), flavonoid content (TFC), and antioxidant activity (DPPH I%) influenced by the study year depending on geographical origin (GO) for each BO of honey.

BO	GO	Year	HMF (mg/kg)	TPC (mg GAE/100 g)	TTC (mg GAE/100 g)	TFC (mg EC/100 g)	DPPH I%
S	AG-C	2021	20.10±0.00 a	146.94±0.04 a	70.56±0.01 a	18.05±0.30 a	21.80±0.03 b
		2022	18.07±0.06 b	126.49±0.02 b	37.41±0.02 b	17.34±0.32 b	33.62±0.04 a
		P	*<0.001*	*<0.001*	*<0.001*	*0.050*	*<0.001*
	AG-G	2021	19.50±0.10 b	275.26±0.11 a	128.13±0.04 a	25.75±0.19 a	27.15±0.01 b
		2022	19.80±0.10 a	121.69±0.03 b	40.09±0.03 b	14.84±0.11 b	30.09±0.04 a
		P	*0.021*	*<0.001*	*<0.001*	*<0.001*	*<0.001*
L	TL-T	2021	34.23±0.50 a	122.57±0.01 a	43.23±0.02 a	12.84±0.12 b	24.70±0.06 b
		2022	19.80±0.10 b	121.69±0.03 b	40.09±0.03 b	14.84±0.11 a	30.09±0.04 a

		P	*0.014*	*<0.001*	*<0.001*	*0.011*	*<0.001*
	GR-B	2021	36.83±0.45 a	99.10±0.02 a	36.81±0.02 a	13.10±0.10 b	36.17±0.04 a
		2022	31.73±0.55 b	96.99±0.03 b	23.53±0.02b	13.35±0.11 a	26.38±0.03 b
		P	*<0.001*	*<0.001*	*<0.001*	*0.044*	*<0.001*
R	AG-C	2021	23.07±0.21 a	104.73±0.02 a	49.52±0.02 a	13.89±0.09 a	23.15±0.03 a
		2022	16.97±0.06 b	97.86±0.04 b	26.14±0.03 b	13.28±0.09 b	21.21±0.06 b
		P	*<0.001*	*<0.001*	*<0.001*	*0.001*	*<0.001*
	TR-B	2021	20.93±0.06 a	107.48±0.04 b	47.57±0.02 a	15.17±0.11 a	38.75±0.15 a
		2022	18.70±0.10 b	117.38±0.03 a	36.68±0.01 b	12.69±0.09 b	20.35±0.03 b
		P	*<0.001*	*<0.001*	*<0.001*	*<0.001*	*<0.001*
	AG-G	2021	23.07±0.21 a	104.73±0.02 a	49.52±0.02 a	13.89±0.09 a	23.15±0.03 a
		2022	16.97±0.06 b	97.86±0.04 b	26.14±0.03 b	13.28±0.09 b	21.21±0.06 b
		P	*<0.001*	*<0.001*	*<0.001*	*<0.001*	*<0.001*
M	TL-C	2021	31.40±0.30 a	109.66±0.04 b	58.82±0.02 a	13.59±0.32 b	20.95±0.03 b
		2022	20.43±0.15 b	110.65±0.03 a	21.68±0.03 b	15.87±0.11 a	25.87±0.04 a
		P	*<0.001*	*<0.001*	*<0.001*	*<0.001*	*<0.001*
	AG-MZ	2021	27.37±0.12 a	168.94±0.04 a	74.33±0.02 a	18.12±0.12 b	23.30±0.02 b
		2022	22.57±0.06 b	112.09±0.01 b	25.76±0.03 b	21.99±0.13 a	29.68±0.03 a
		P	*<0.001*	*<0.001*	*<0.001*	*<0.001*	*<0.001*
A	AG-C	2021	16.63±0.06 a	164.79±0.01 a	117.12±0.05 a	13.63±0.30 a	13.98±0.02a
		2022	15.80±0.10 b	72.59±0.04 b	38.11±0.01 b	10.69±0.34 b	12.73±0.06 b
		P	*<0.001*	*<0.001*	*<0.001*	*<0.001*	*<0.001*
	AG-V	2021	16.10±0.10 a	80.70±0.02 a	63.40±0.02 a	11.72±0.08 a	23.02±0.03 b
		2022	15.53±0.06 b	60.19±0.02 b	33.50±0.01 b	10.63±0.08 b	29.23±0.04 a
		P	*0.001*	*<0.001*	*<0.001*	*<0.001*	*<0.001*
	AG-MO	2021	18.40±0.10 a	76.11±0.01 b	57.02±0.03 b	11.49±0.08 b	20.08±0.08 a
		2022	13.93±0.06 b	86.70±0.04 a	67.24±0.02 a	12.82±0.09 a	18.35±0.04 b
		P	*<0.001*	*<0.001*	*<0.001*	*<0.001*	*<0.001*

Mean±SD from all geographical origins is presented. Means with the same letter in each column are not significantly different at a 5% level, in accordance with Duncan`s Multiple Range Test.
P= significance of the year's influence, presented in accordance with the One-Way Analysis of Variance (at a significance level of α=.05).
S= sunflower honey, L= linden honey, R= rapeseed honey, M= multifloral honey, A= acacia honey.
AG-C=Arges-Costesti, AG-G=Arges-Gliganu, AG-MO=Arges-Mosoaia, AG-MZ=Arges-Mozaceni, AG-V=Arges-Vedea, GR-B=Giurgiu-Bolintin, TR-B=Teleorman-Branceni, TL-C=Tulcea-Casimcea, TL-T=Tulcea-Topolog.

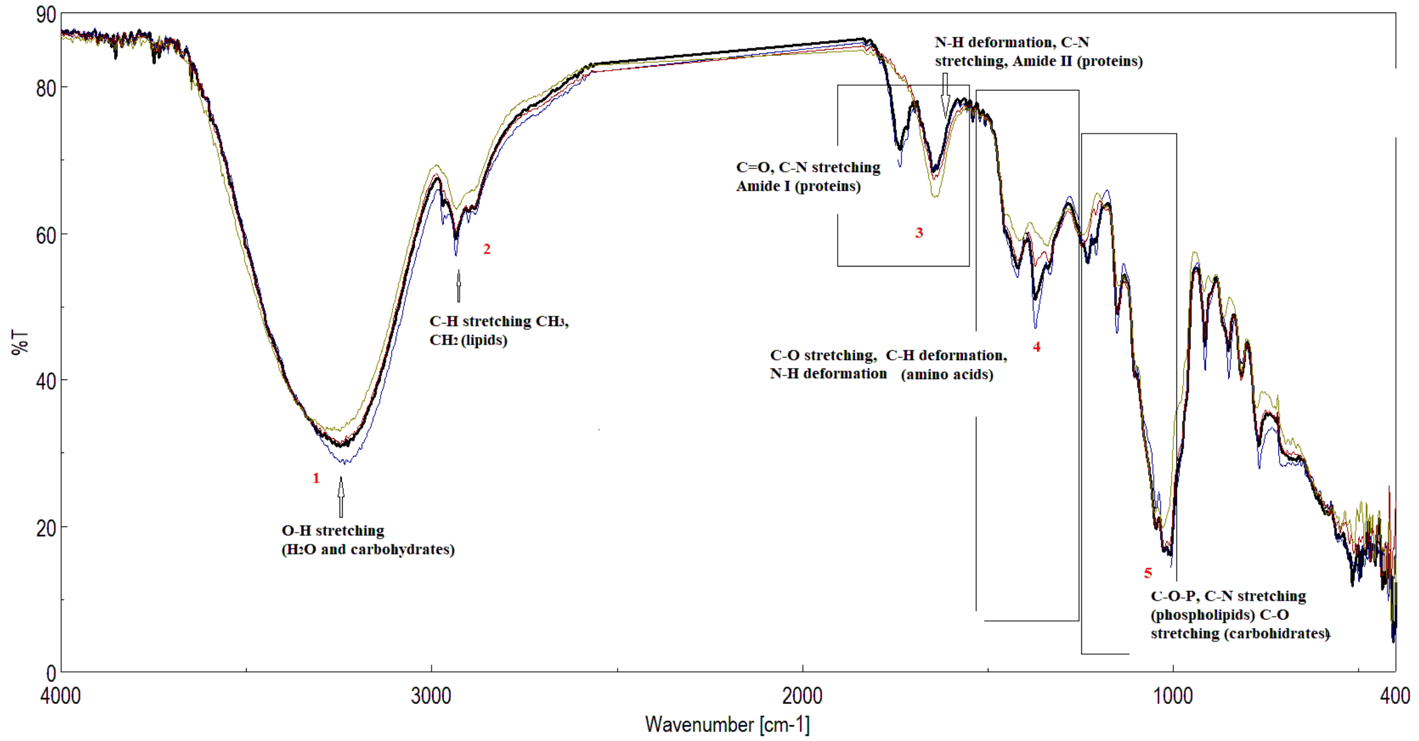

Figure S1. Representative ATR-FTIR spectrum of sunflower honey (1 - stretching vibrations of OH, 2 - C-H stretching vibrations of CH_3 and CH_2 from lipids, 3 and 4 - C=O, amide I, amide II, C-N from proteins, 5 - C-O stretching in carbohydrates, phosphate band).
